# Supplementary material for: A multiplexed DNA FISH strategy for assessing genome architecture in Caenorhabditis elegans
Source: eLife. 2019 May 14;8:e42823. doi: 10.7554/eLife.42823 (PMC6516958; doi:10.7554/eLife.42823)
Supplement: Supplementary file 6. [file elife-42823-supp6.docx]

**T7 Oligopaint Synthesis –Protocol**

(Adapted from MERFISH protocol, Zhuang lab, Chen et al 2015 Science)

**Overview of the method:**

1) Amplification of the library, low cycle (~25 cycles)

2) Another amplification round from the product from step 1, and using touch-up PCR to add the T7 promoter to the reverse strand

3) T7 RNA synthesis (overnight incubation)

4) Reverse transcriptase for single-stranded DNA probe synthesis

5) Degradation of RNA via alkaline hydrolysis

6) Purification of ssDNA

**“Linear” PCR of Library or Sub-library**

· Goal: since the amount of “raw” library is limited (ie. CustomArray produces around 80 ul of a library at around 50-100 ng/ul), we regularly perform PCR to produce more template

Materials:

□ DNA Clean and Concentrator Kit -5 (Zymo D4013 or D4014)

□ Library (ie. Customarray 90K Oligo Pool)

□ Taq (Kapa BK1002)

□ dNTPs (10 mM stock)

| **Reagent** | **Initial** | **Final** | **1 reaction** |
| --- | --- | --- | --- |
| Kapa Buffer A | 10x | 1x | 10 ul |
| Forward Primer | 200 uM | 2 uM | 1 ul |
| Reverse Primer | 200 uM | 2 uM | 1 ul |
| dNTP mix | 10 mM | 0.4 mM | 4 ul |
| Kapa Taq* | 5 U/ul | 5 U | 1 ul |
| Raw library |  | 10-30 ng | 1 ul |
| Water (**up to 100 ul**) |  |  | 82 ul |

*you can opt to use different Taq if you wish, but we generally use Kapa Taq for our experiments

1) Linear PCR program **-**95C-5min, (95C-30s, **58C**-30s, 72C-15s)X**25**, 72C-5min

2) Purify with Zymo-5 kit, following manufacturer’s instructions

a. We also add an additional spin step after last wash (max speed, no buffer) to remove residual ethanol from wash buffer

3) resuspend in ~30 ul of water

4) Expected yield: ≥ 20 ng/ul

**Bulk touch-up PCR – to add T7 promoter**

· Goal: produce PCR product with the T7 promoter, enough to produce 500 ng – 1 ug of product

Materials

□ Reverse Primer with T7 promoter sequence

o Add TAATACGACTCACTATAGGG to the 5’ end of the reverse primer

o Note that T7 synthesis later adds the last three Gs into the sequence

1) Make the following master mix

a. Here we are doing a 5x reaction so that we have enough template to do multiple T7 reactions

| **Reagent** | **Initial** | **Final** | **1 reaction** | **5 reactions** |
| --- | --- | --- | --- | --- |
| Kapa Buffer A | 10x | 1x | 10 ul | 50 ul |
| Forward | 200 uM | 0.4 uM | 0.2 ul | 1 ul |
| Reverse (T7) | 200 uM | 0.4 uM | 0.2 ul | 1 ul |
| dNTP mix | 10 mM | 0.2 mM | 2 ul | 10 ul |
| Kapa Taq |  |  | 1 ul | 5 ul |
| Linear-amplified library | 1 ng/ul | 0.5 ng | 0.5 ul | 2.5 ul |
| Water (**up to 100 ul**) |  |  | 86.1 ul | 431 ul |
|  |  | Total | 100 ul |  |

***NOTE I have also used Q5 polymerase reaction for this step and it works well.

2) Do new program (based on EH1) T7PCR35 -95C-5min, (95C-30s, 60C-30s, 72C-15s)X35, 72C-5min

3) Purify – using PCR purification kit from qiagen, elute in 30 ul of water

4) **Expected Result:** you want samples to be around 200-300 ng/ul or more

**T7 Reaction (using manufacturer's suggestions)**

· Goal: produce excess RNA in order to maximize the amount of dNTPs

- 1 reaction can produce around 750 to 1500 pmol of probe

Materials

□ T7 HiScribe Kit (NEB E2040S)

□ Zymo-100 DNA Clean and Concentrator Kit (D4029 or D4030)

□ RNAseOUT (Thermo, 10777019)

1) Measure DNA to make sure amplification worked , run on gel

2) Use 1 ug of DNA per reaction

a. While you may opt to do 1 reaction, for large scale probe synthesis you can fit 4x reactions comfortably in a 1.5 ml tube

b. If you decide on the larger format (1.5 ml tube), make sure you have proper equipment to do incubations later on (at 37 ^o^C, 50 ^o^C, 85 ^o^C, and 95 ^o^C)

| **Reagent** | **1x reaction**  **(manufacturer)** | **4x reactions** |
| --- | --- | --- |
| DNA | 3 ul (assuming ~300 ng/ul) | 12 ul |
| Water | 7 ul | 28 ul |
| ATP (provided by kit) | 1.5 ul | 6 ul |
| CTP (provided by kit) | 1.5 ul | 6 ul |
| GTP (provided by kit) | 1.5 ul | 6 ul |
| UTP (provided by kit) | 1.5 ul | 6 ul |
| T7 buffer | 1.5 ul | 6 ul |
| RNaseOUT | 1 ul | 4 ul |
| T7 Pol Mix | 1.5 ul | 6 ul |
|  | 20 ul | 80 ul |
|  |  |  |

Split into four 20 ul reactions and incubate in a PCR machine overnight at 37C.

**RT PCR:**

· Goal: convert RNA to DNA

Materials

□ Forward Primer

□ dNTPs (25 mM stock)

□ maxima RT –H (Thermo, EP0751)

□ RNAseOUT (Thermo, 10777019)

1) No need to purify the T7 sample; add RT reagents right into tube and incubate

| **Reagent** | **Initial** | **Final** | **1 reaction** | **4 reactions** |
| --- | --- | --- | --- | --- |
| T7 sample |  |  | 20 ul | 80 ul |
| Water |  |  | 79.4 ul | 317.6 ul |
| Forward primer | 200 uM | 1500 pmol | 7.5 ul | 30 ul |
| dNTP mix | **25 mM** | 1.6 mM | 9.6 ul | 38.4 ul |
| RT buffer | 5x | 1x | 30 uL | 120 ul |
| RNaseOUT |  |  | 1.5 uL | 6 ul |
| Maxima RT -H |  |  | 2 uL | 8 ul |
|  |  | Total | 150 uL | 600 ul |

2) Incubate for **two hours** at 50 ^o^C (this is longer than manufacturer suggestion)

3) Inactivate RT at 85 C for 5 min

**RNA degradation via alkaline hydrolysis:**

· Goal: removal of RNA from the RNA:DNA hybrids, and removal of excess RNA not converted to cDNA

Materials

□ EDTA (0.5 M)

□ NaOH (1 M)

1) Digest remaining RNA:

a. by mixing 0.5 EDTA and 1 M NaOH, 1:1 to each other, and then add to RT reaction at equal volume (final conc: 0.25 M NaOH)

i. 1x reaction: add 150 ul of EDTA-NaOH mixture to each sample

ii. 4x reaction: add 600 ul

b. Heat at 95 ^o^C for 10 min

i. Alternative alkaline hydrolysis temperatures and times may also work

c. Put on ice and proceed right away with purification

d. Total rxn vol:

i. 300 ul for 1x reaction

ii. 1200 ul for 4x reaction

**Purification with Zymo-100 kit**

Materials

□ Zymo-100 DNA Clean and Concentrator Kit (D4029 or D4030)

o We use this kit because of the scalability to purify 100 ug of DNA, but because the oligos are single stranded and short, we DO NOT use DNA binding buffer

o Instead, we use Oligo binding buffer (purchased separately)

□ Oligo Binding Buffer, 40 ml (D4060-1-40)

□ Wash Buffer (additional bottles - D4003-2-48)

1) Add Oligo binding buffer (ordered separately from Zymo) (2x rxn vol = 2400 ul)

2) Add 100% Ethanol (4x oligo binding buffer = 9600 ul)

3) Purify on column and follow manufacturer instructions

**Calculate Yield**

1) ng/ul to pmol/ul conversion:

o Formula: ( ng/ul concentration) * (1E3 pg/ ng) * (1 pmol nucleotides / 330 pg) * (1 /length in nucleotides)

o [conc] * 3.03 * (1/length) = ____ pmol/ul

2) Total pmol amount - Multiply concentration to elution volume

3) Calculate yield by: probe (pmol)/RT primer (pmol) * 100

o We generally get 50% yield or more
