# Supplementary material for: A multiplexed DNA FISH strategy for assessing genome architecture in Caenorhabditis elegans
Source: eLife. 2019 May 14;8:e42823. doi: 10.7554/eLife.42823 (PMC6516958; doi:10.7554/eLife.42823)
Supplement: Supplementary file 7. [file elife-42823-supp7.docx]

**Equipment:**

2 Heat blocks (65°C and 95°C/100°C)

Hybridization oven (37°C)

Rocker/Rotator (room temperature)

Bench top centrifuge (all spins for this protocol are at 3,000 rpm for 30 seconds)

Aluminum foil

Microscope slides

Cover slips (22x22, no. 1.5)

Hybridization chamber (enclosed plastic container with damp paper towel)

1.5 ml microcentrifuge tubes (Fisher Scientific: 02-682-002)

Superfrost plus gold slides (Thermo Scientific: FT4981GLPLUS). Note: only for embryo isolation protocol

Needles or Scalpels (only for embryo isolation protocol)

Parafilm (only for embryo isolation protocol)

**Reagents:**

100% Ethanol (Koptec: V1001G)

10X PBS (Thermo Fisher Scientific: 70011-044)

Triton X-100 (Sigma: X100)

Tween-20 (Sigma: P1379)

16% Paraformaldehyde ampoules (Electron Microscopy Sciences: 15710)

Formamide (Millipore: 344206)

20X SSC (Thermo Fisher Scientific: 15557-044)

Dextran sulfate (Sigma: 42867)

RNAse A (Sigma: R6148)

Mounting Media (Vectashield with DAPI (Vector-H-1200) or Slowfade Gold with DAPI (Thermo Fisher Scientific-S36938) NOTE: Make sure the mounting media is compatible with the chosen fluorophores.

Primary Oligopaint oligos concentrated to 100 pmol/ul

Stock solutions of bridge and detection oligos stored at 200 pmol/ul

Liquid nitrogen

Bleach Solution

M9 Solution (for worm collection)

Egg buffer (only for embryo isolation protocol)

**Buffers and solutions:**

**1XPBST**

500 ml working solution:

50 ml 10X PBS

447.5 ml distilled H2O

2.5 ml Triton X-100

**Formaldehyde fix solution**

40 ml working solution:

4 ml 10X PBS

26 ml distilled H2O

10 ml 16% paraformaldehyde

**2XSSC**

500 ml working solution:

50 ml 20X SSC

450 ml distilled H2O

**2XSSCT**

500 ml working solution:

50 ml 20X SSC

447.5 ml distilled H2O

2.5 ml Triton X-100

**95% ethanol (keep cold at -20°C)**

40 ml working solution:

47.5 ml 100% ethanol

2.5 ml distilled H2O

**50% formamide 2X SSC (make fresh each day)**

10 ml working solution:

5 ml formamide

1 ml 20X SSC

4 ml distilled H2O

**Hybridization solution:**

50 ml solution:

20 g Dextran Sulfate

20 ml 20X SSC

250 ul Tween-20

Distilled water up to 50 ml

**Primary hybridization mix (make fresh each experiment)**

60 ul total:

100 pmol of each primary probe

2 ul RNAse A

Water to 15 ul

30 ul formamide

15 ul hyb solution

**Bridge and detection oligo hybridization mix**

60 ul total:

15 ul bridge oligo solution (100 pmol of each bridge oligo per structure)

For example, if imaging a single chromosome use 25 pmols of each of the 4 bridge oligos in 15 ul of H2O.

15 ul detection oligo solution (100 pmol of each detection oligo in H2O)

18 ul formamide

12 ul distilled H2O

**M9 Solution**

500 ml working solution:

1.5 g KH2PO4

3 g Na2HPO4

0.25 g NaCl

0.5 g NH4Cl

Bring to 500 ml with distilled H2O

5 ul of Triton X-100 to inhibit worms from sticking to plastic

**Bleach Solution**

100 ml working solution:

2.5 ml NaOH (50% in H2O)

20 ml Germicidal bleach (Clorox brand-8.25% Sodium Hypochlorite)

77.5 ml H2O

**Egg buffer (only for embryo isolation protocol)**

50 ml 10X Stock solution:

12.5 ml 1M HEPES, ph 7.4

11.8 ml 5M NaCl

24 ml 1M KCl

100 ul 1M CaCl

100 ul 1M MgCl

1.5 ml distilled water

**Sample collection for whole animal Oligopaint:**

The goal is to collect a 30-50 ul pellet of *C. elegans* and snap freeze in liquid nitrogen. If mixed stage animals are desired grow a mixed stage population on 10 cm plates seeded with OP50 bacteria.

1. Wash off animals with 10 ml of M9 solution.

2. Spin down at 3000 rpm for 30 seconds, remove supernatant. NOTE that all spins for this protocol are 3000 rpm for 30 seconds.

3. Add fresh M9 solution and let rock for ~30 minutes at room temperature.

4. Spin down again and remove excess M9.

5. Aliquote 30-50 ul pellets of worms into 1.5 ml microcentrifuge tubes.

6. Place in liquid nitrogen for 1 minute and store at -80C.

If only adult animals are desired begin by isolating embryos using hypochlorite treatment:

1. Collect adult worms from a 10 cm plate in M9

2. Spin down and resuspend in 6 ml of bleach solution.

3. Shake by hand for 5 minutes, spin down, and wash twice with M9 solution.

4. Place ~5,000 embryos onto a fresh 10 cm plate seeded with OP50

5. Once worms are adults, continue with sample prep as described above.

**Oligopaint FISH on intact animals:**

Things to do before starting:

Set heat blocks to 65°C and 95°C, set hybridization oven to 37°C, and make sure 95% ethanol is chilled at -20°C. If desired one can make hybridization mixtures right before starting.

1. Resuspend frozen pellet in 1 ml cold 95% ethanol and vortex on high for 1 minute

2. Rock for 9 additional minutes at room temperature

3. Spin down, remove ethanol, and resuspend in 1 ml 1X PBST

4. Repeat wash

5. Resuspend pellet in 1 ml of formaldehyde fix solution

6. Rock at room temperature for 6 minutes

7. Spin down, remove fix, and resuspend in 1 ml 1X PBST

8. Repeat wash

9. Resuspend in 1 ml 2X SSC, let sit at room temperature for 5 minutes

10. Spin down, resuspend in 50% formamide 2X SSC solution

11. Let sit at room temperature for 5 minutes

12. Transfer to 95°C heat block for 3 minutes

13. Transfer to 65°C heat block for 20 minutes

14. Use the 20 minutes to prepare hybridization mix

15. Spin down, remove excess solution

16. Repeat spin to remove excess solution

17. Resuspend sample in 60 ul of primary hybridization mix

18. Pipette slowly up and down to mix the sample well

19. Transfer to 100°C heat block for 5 minutes

20. Wrap tubes together in aluminum foil and place inside hybridization oven overnight (at least 12 hours)

Next Day

~1 hr before you start prewarm the hybridization oven, 2X SSC, and 2X SSCT to 60°C. Note that our lab has one hyb oven, so I transfer the samples to a 37°C incubator in the meantime.

21. Add 1 ml of warm 2X SSCT to the worms/hybridization mix, spin down, remove excess

22. Resuspend in warm 2X SSCT, rotate in 60°C hybridization oven for 5 minutes

23. Spin down, remove excess, and resuspend in warm 2X SSCT

24. Rotate at 60°C for 20 minutes

25. Spin down, remove excess, repeat again.

26. Resuspend in 60 ul of bridge/detection oligo hybridization mixture

27. Place in the dark at room temperature for 3 hours.

28. Spin down and remove excess

29. Resuspend in warm 2X SSC

30. Place in 60°C hybridization oven for 20 minutes

31. Spin down and remove excess

32. Resuspend in warm 2X SSCT

33. Place in 60°C hybridization oven and rotate for 5 minutes

34. Spin down and remove excess

35. Resuspend in warm 2X SSCT

36. Place in 60°C hybridization oven and rotate for 20 minutes

37. Spin down and remove excess

38. Resuspend in 2X SSCT and rock at room temperature for 5 minutes (keep covered from light)

39. Spin down and remove excess.

40. Repeat to remove as much as possible.

41. Resuspend in 50 ul of mounting media

42. Place 15 ul of sample in mounting media onto a 22X22 coverslip

43. Sandwich gently onto a microscope slide

44. Seal with nail polish

**Oligopaint FISH on isolated embryos:**

Before starting: Pre chill 95% ethanol at -20°C, and set heat block to 90°C. Note that a flat surface is needed to place the slide on the heat block, so if possible flip over heat block to use the flat bottom. Set hybridization oven to 37°C.

1. Pick ~20 adult animals into 8 ul of egg buffer

2. Dissect animals using a needle or scalpel. Cut near the middle of the animal to release embryos

3. Place coverslip onto a Superfrost Plus Gold slide

4. Using tongs, submerge slide in liquid nitrogen for 1 minute

5. Remove from liquid nitrogen and quickly pop off coverslip, submerge in 95% ethanol at -20°C for 10 minutes. Note: this step must be done QUICKLY.

6. Wash twice with 1X PBST

7. Add 100 ul of fixation solution directly onto sample

8. Cover with a coverslip sized piece of parafilm and place in hybridization chamber for 5 minutes.

9. Wash twice with 1X PBST

10. Add 20 ul of primary hybridization mix directly to sample and cover with a coverslip

11. Place slides on the flat side of a 90°C heat block for 10 minutes.

12. Transfer slides to a pre-warmed hybridization chamber and place hybridization chamber in hybridization oven.

13. Let sit overnight (at least 12 hours)

Next Day:

~1 hr before you start prewarm the hybridization oven, 2X SSC, and 2X SSCT to 60°C. Note that our lab has one hyb oven, so I transfer the samples to a 37°C incubator in the meantime.

14. Wash slide in warm 2X SSCT for 5 minutes at 60°C

15. Repeat wash with warm 2X SSCT for 20 minutes at 60°C

16. Wipe off excess wash buffer and place 20 ul of secondary/detection hyb mix onto sample. Place coverslip on top and incubate at room temperature for 3 hours in the hybridization chamber (keep away from light)

17. Wash with warm 2X SSC for 20 minutes at 60°C

18. Wash with warm 2X SSCT for 5 minutes at 60°C

19. Wash with warm 2X SSCT for 20 minutes at 60°C

20. Wash with 2X SSCT for 5 minutes at room temperature (keep away from light)

21. Wipe off excess wash buffer and add 15 ul of mounting media

22. Place coverslip on top

23. Seal with nail polish
