# Supplementary material for: A multiplexed DNA FISH strategy for assessing genome architecture in Caenorhabditis elegans
Source: eLife. 2019 May 14;8:e42823. doi: 10.7554/eLife.42823 (PMC6516958; doi:10.7554/eLife.42823)

The goal of this analysis workflow is to determine both the volume of chromosome territories and the amount of overlap between chromosome territories (specifically chromosomes I, II, and III).

Software:

FIJI/ImageJ (<https://imagej.net/Fiji/Downloads>)

Plugins required:

3D Objects counter (https://imagej.net/3D_Objects_Counter)

3D Manager (<http://imagejdocu.tudor.lu/doku.php?id=plugin:stacks:3d_roi_manager:start>)

1. Segment nuclei from the image to generate individual nuclei files. Using the rectangle tool in imageJ, draw a box around the nuclei of interest. For this image the 3 chromosomes are represented by 3 individual channels (green, red, blue representing chromosomes I, II, and III respectively.) Go up and down in Z to determine the Z slices that contain fish signal for the given nuclei. Use the duplicate function (Image > Duplicate), choose “duplicate hyperstack”, the range of z slices containing signal, and the channels of interest.


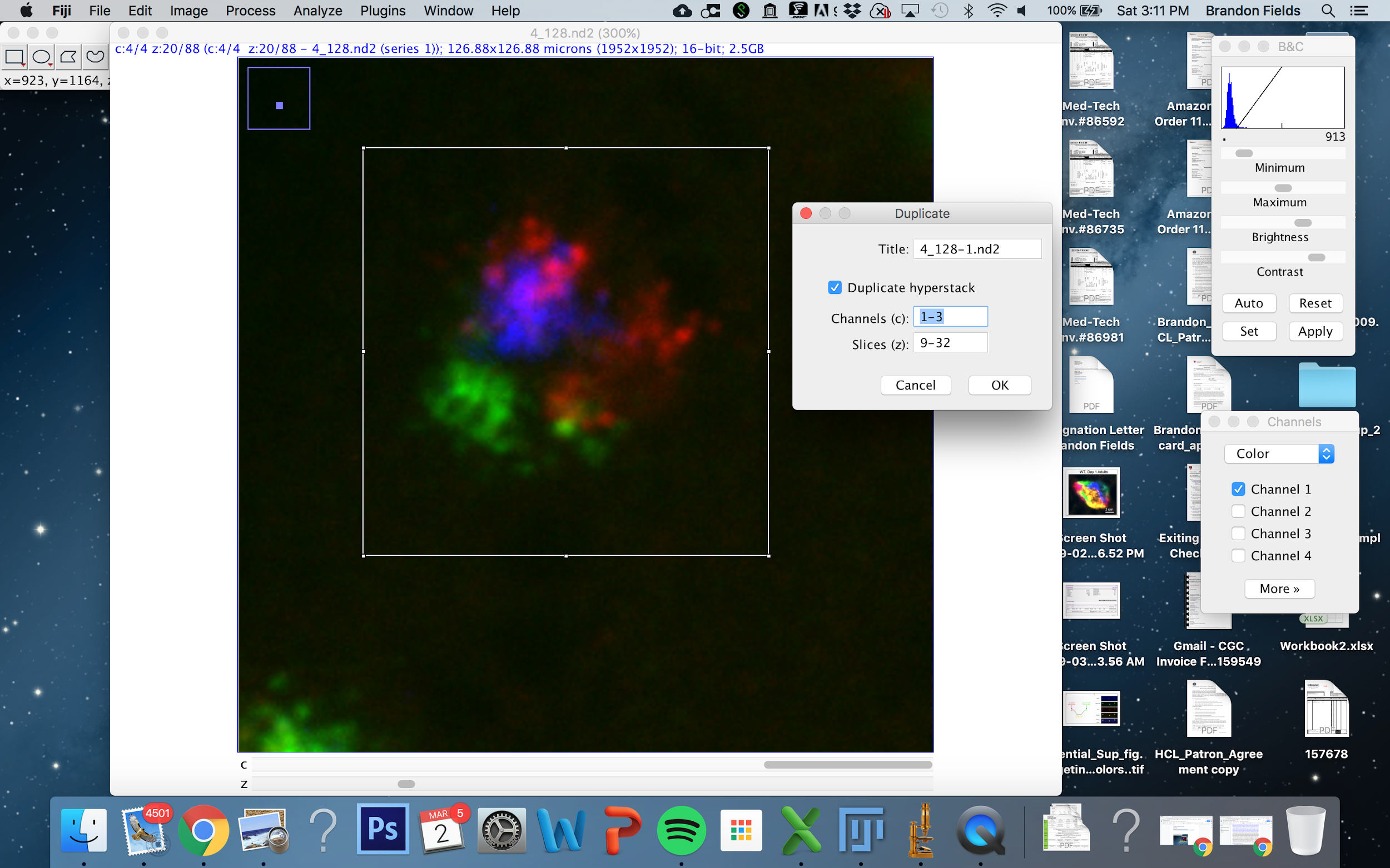


2. The output of Step 1 is a 3 channel image of a single nuclei (below)


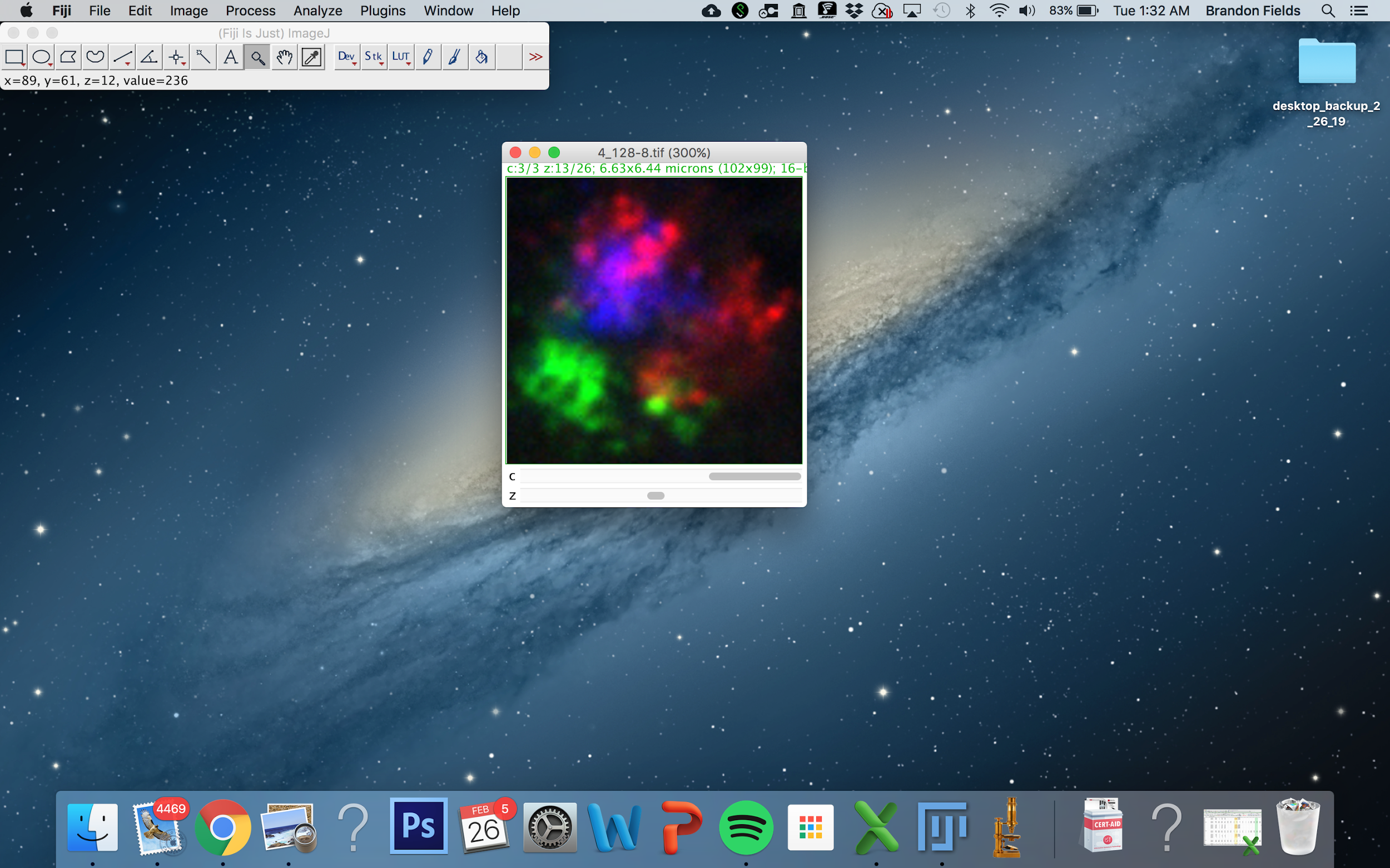


2. Split the image to generate three independent channels (Image > Color > Split channels)


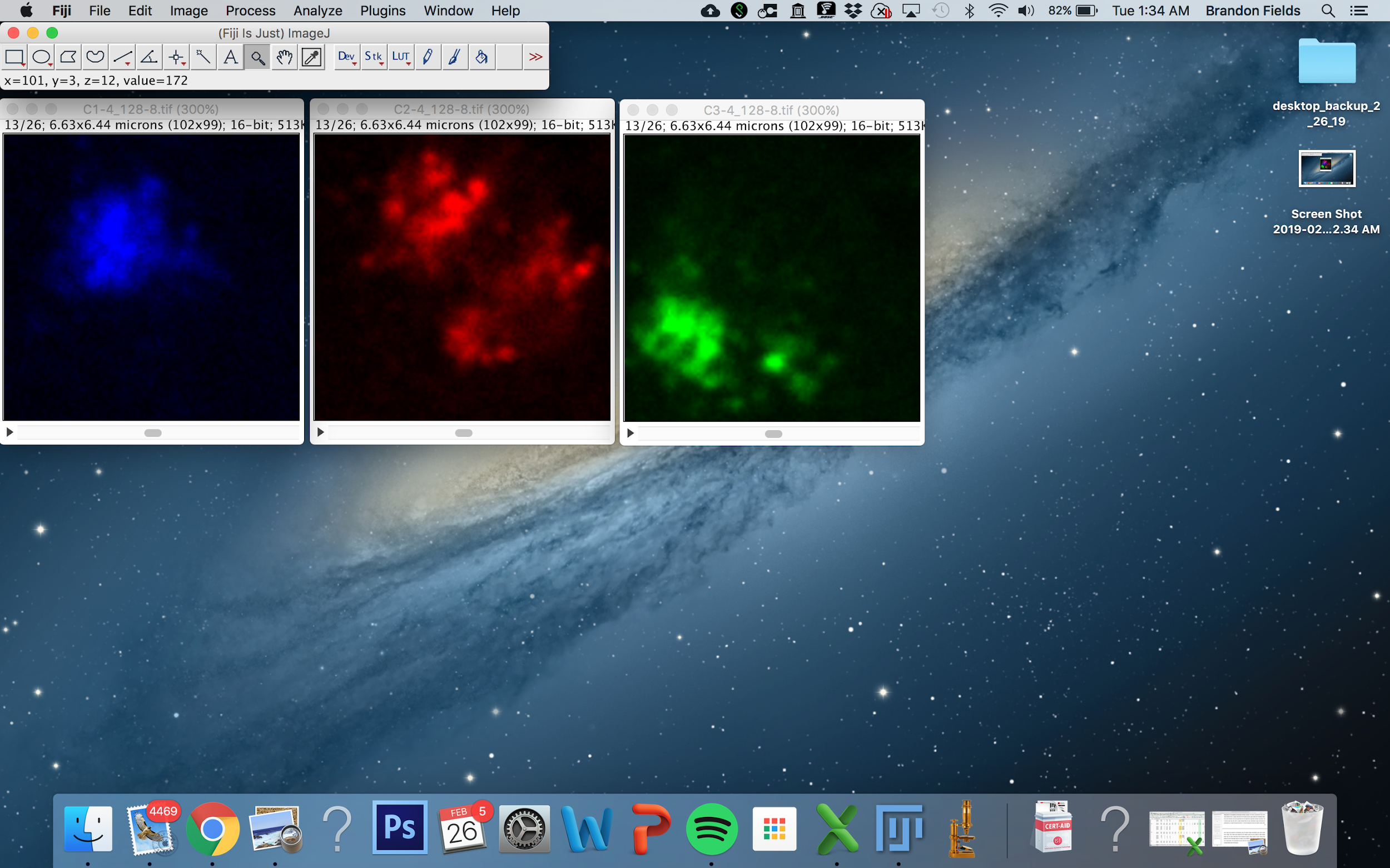


3. Next the computer needs to know what part of the image is FISH signal via thresholding. For a single channel go to a z slice that contains FISH signal. Open the threshold window (Image > Adjust > Threshold), and choose “auto”. This will automatically determine the threshold value for FISH signal vs background. Note that different thresholding algorithms exist and “default” was used for this study. Press Apply.


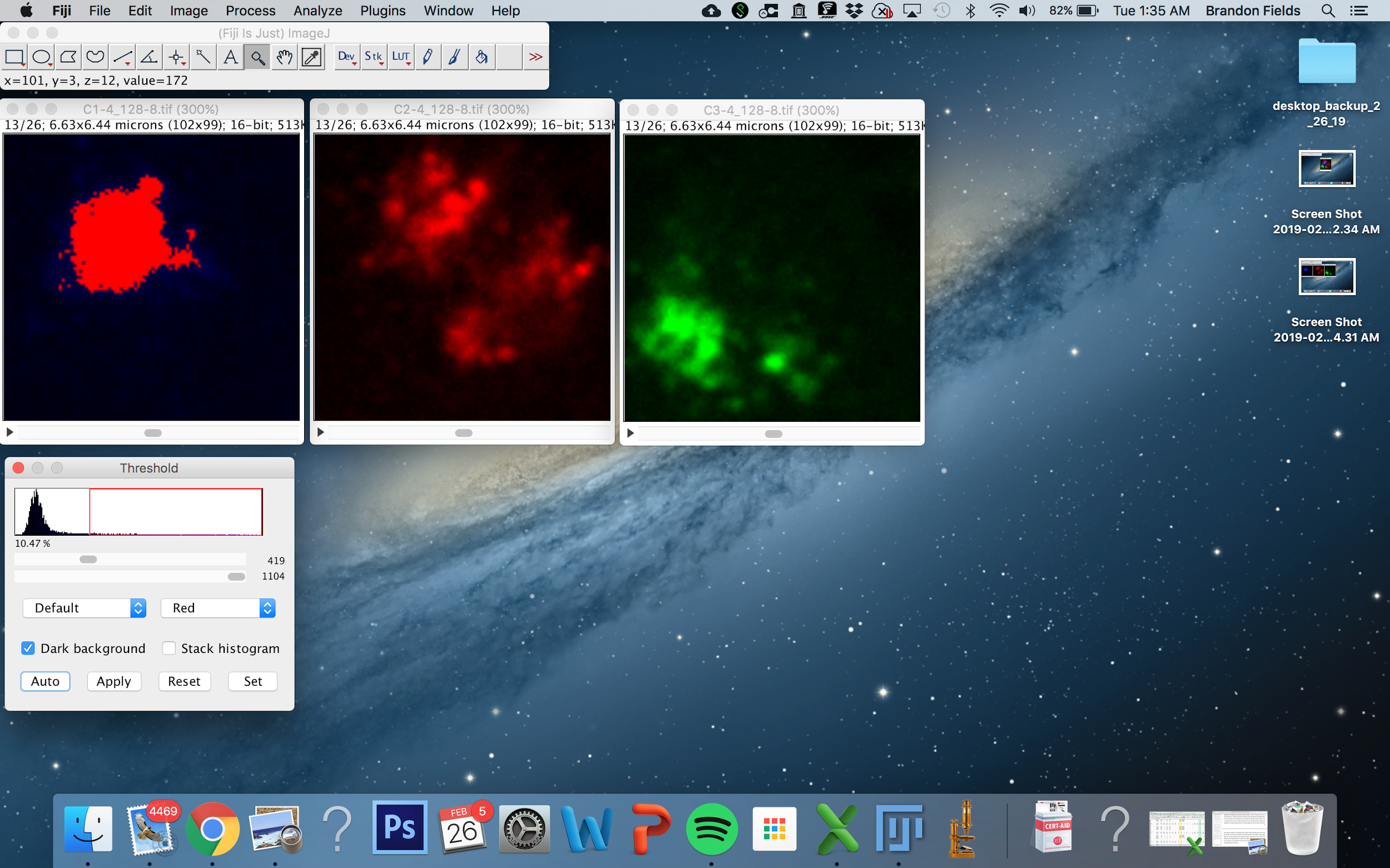


4. Uncheck “Calculate threshold for each image”. This will use the value determined in Step 3 for all z slices. Removing this option was done to reduce background signals from out of focus light at the top and bottom most Z slices.


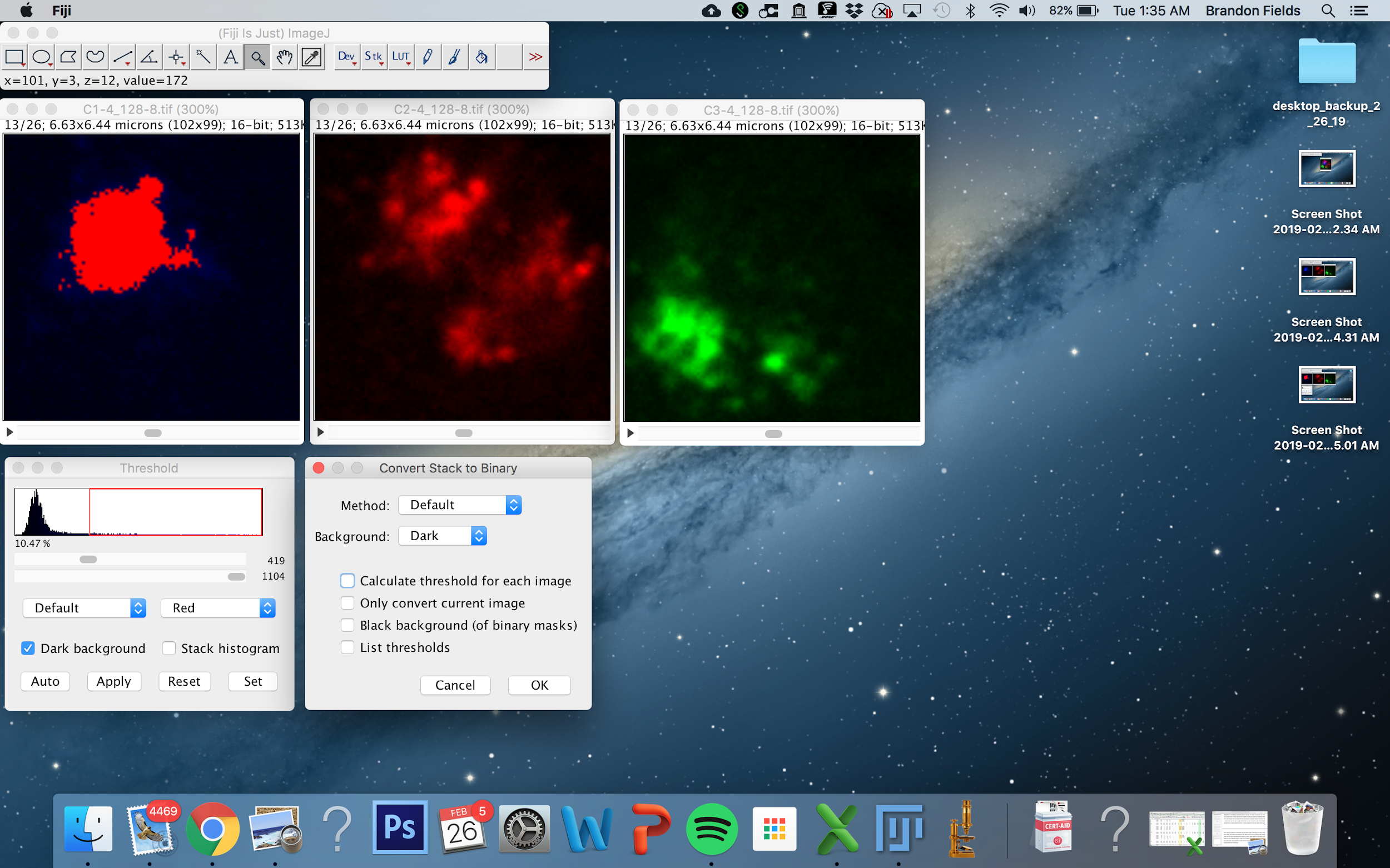


5. The result of Step 4 is a binary image where black represents the FISH signal:


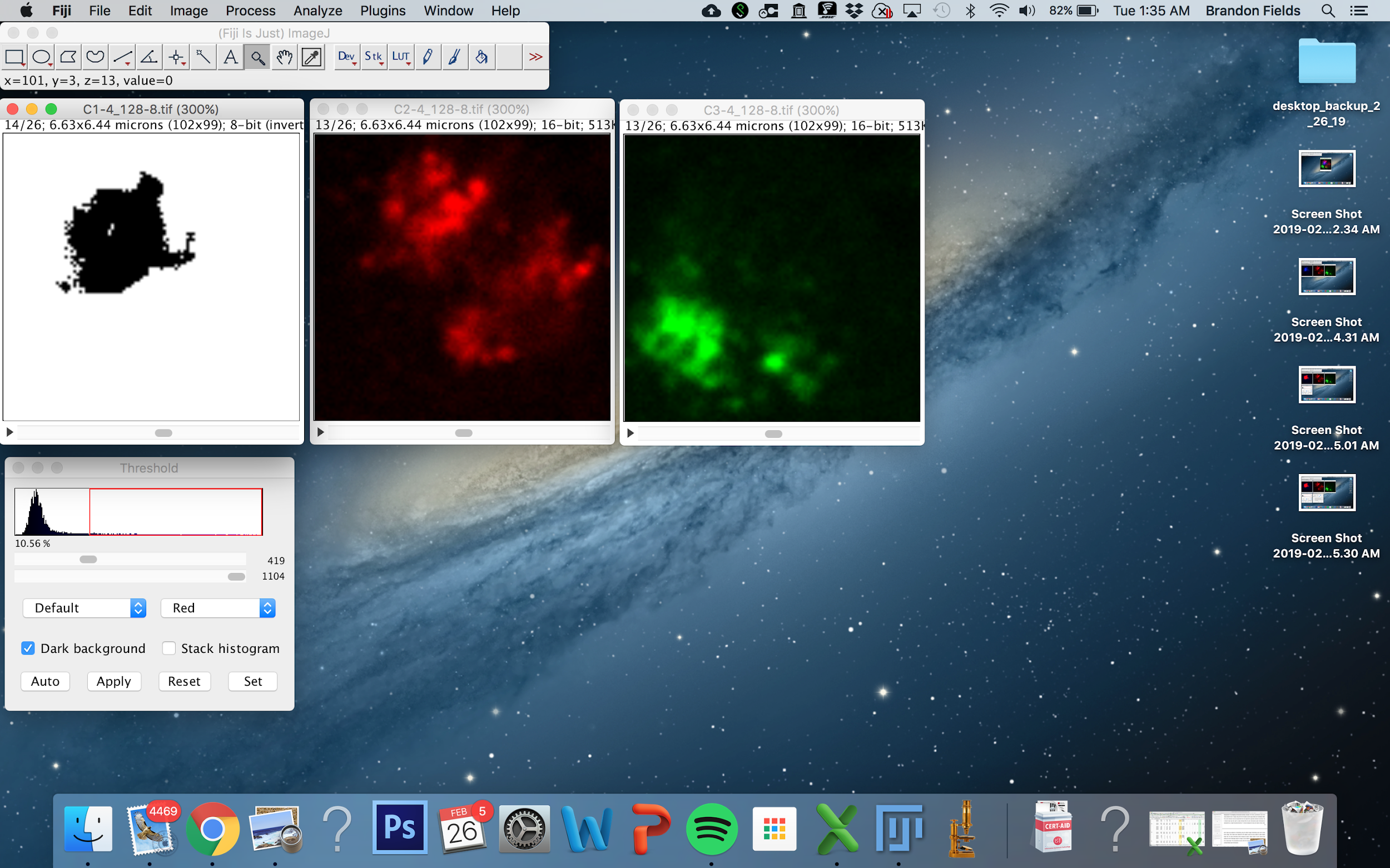


6. Repeat steps 3-5 for the two other channels in the image:


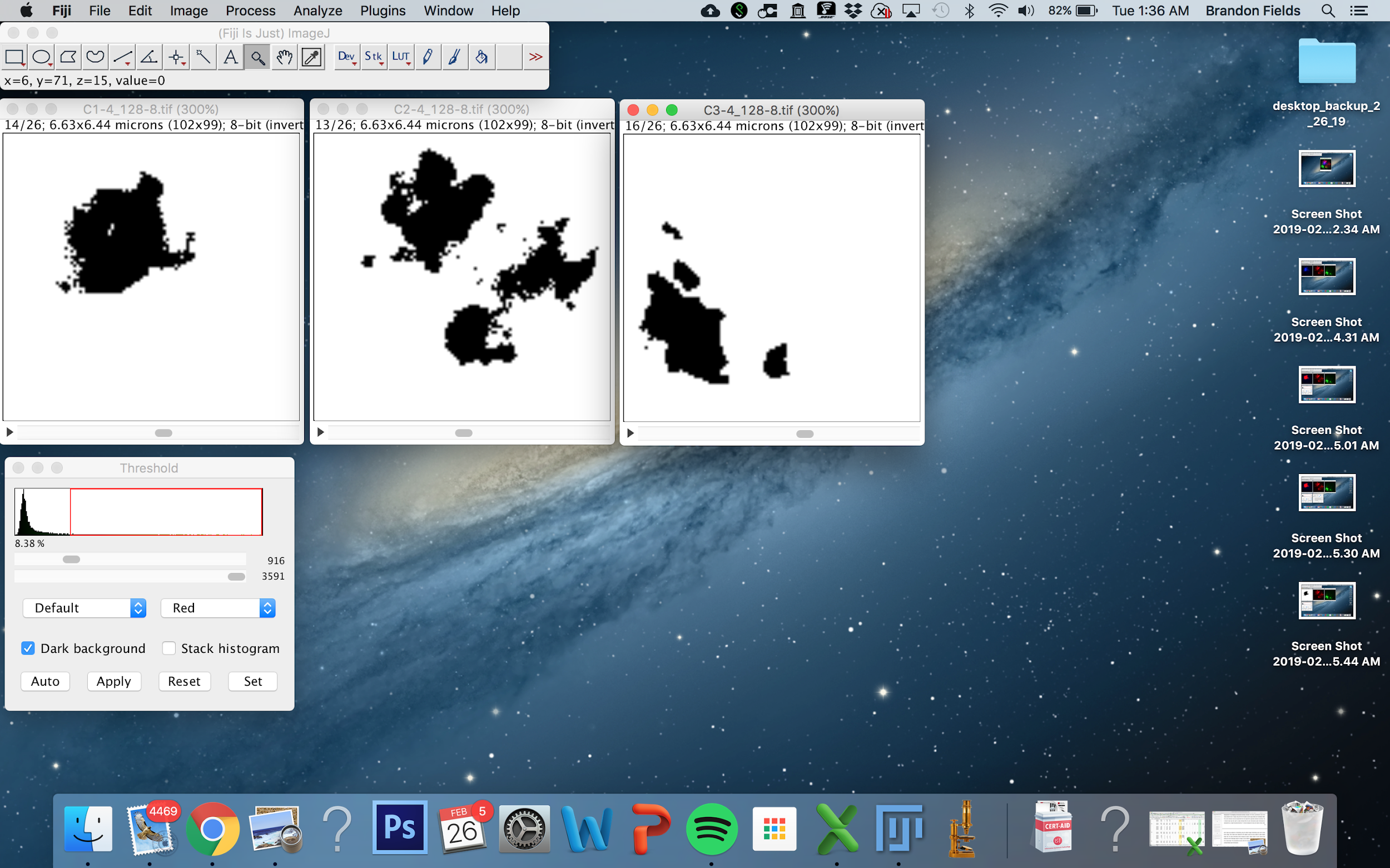


7. Next we will use the 3D Objects counter plug in counter to generate 3D object maps for each of the channels:


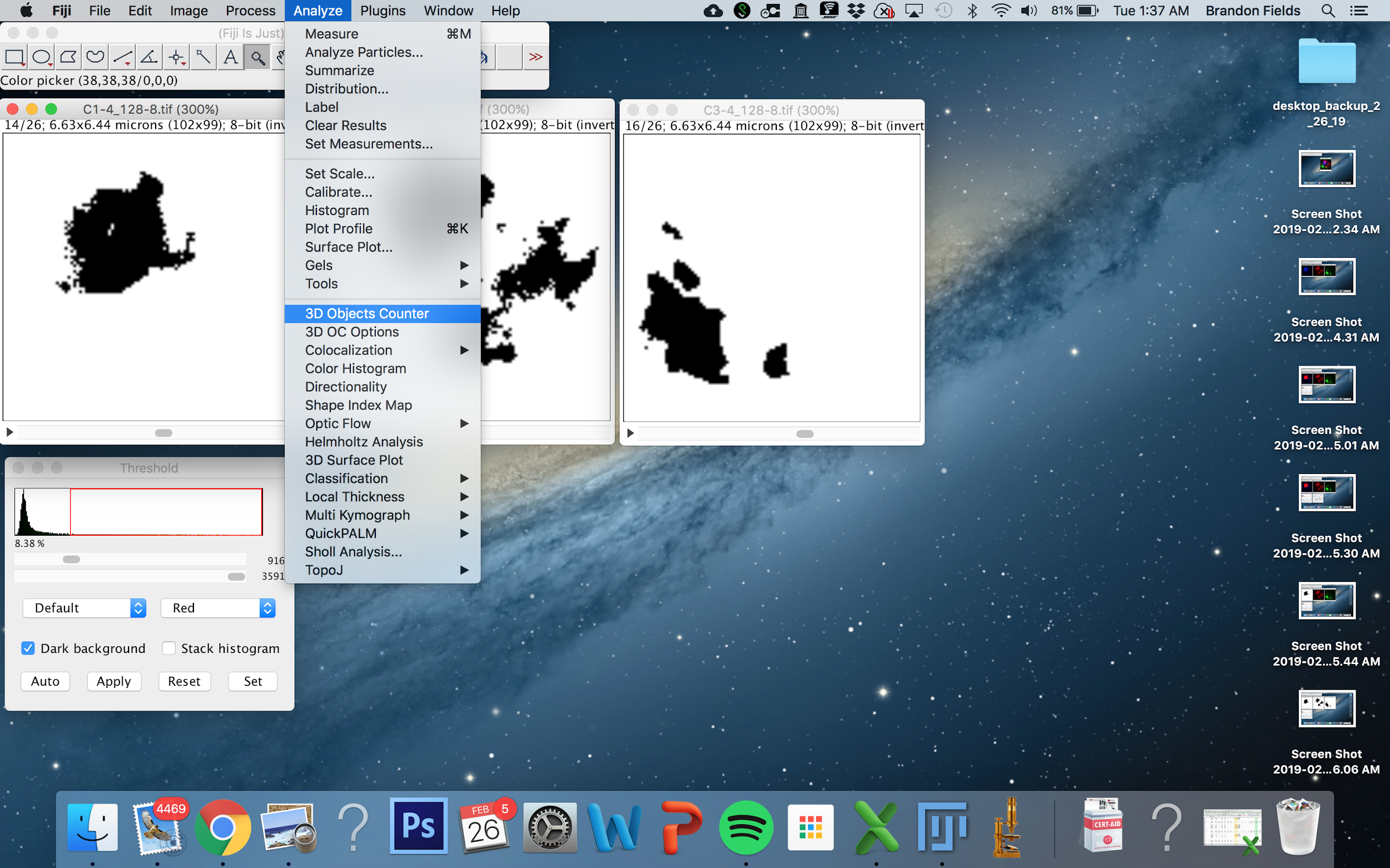


8. Choose “Objects” under maps to show, and “Statistics” for result tables to show. For this study the min volume size was put at 30 voxels to remove background pixels of high intensity and used across all images. This value can be changed to increase stringency (increase the min) or decrease stringency (decrease the min):


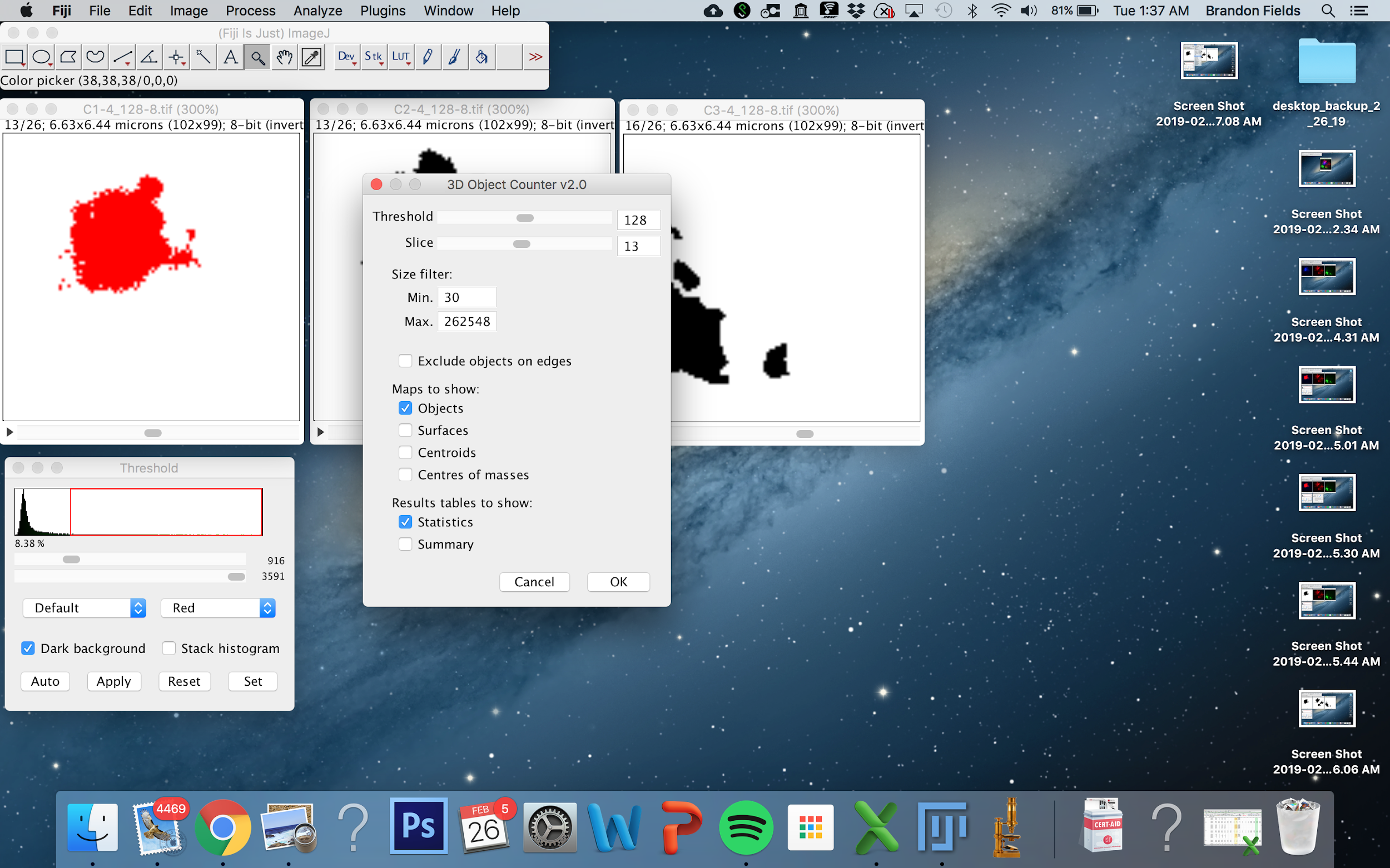


9. Save both the objects map files and the statistics file for each channel. Note that the object map files will be required in the next step, whereas the statistics file is not absolutely required but is nice to have if you want to go back and look at various statistics. Repeat for the other two channels:


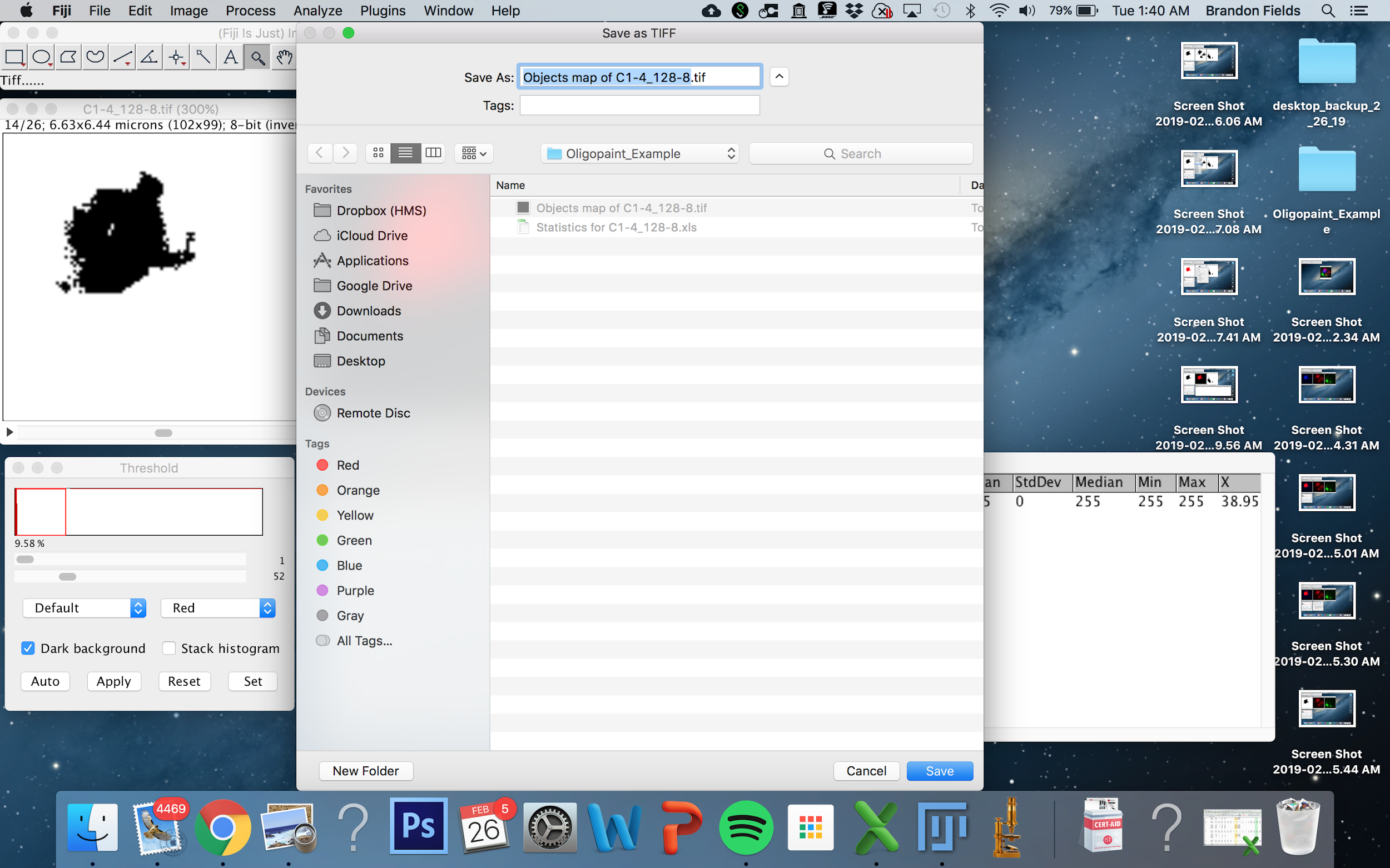


10. Close all open files and open each of the 3 objects maps files. Note that each of the detectable “objects” within a channel will be represented by a different color:


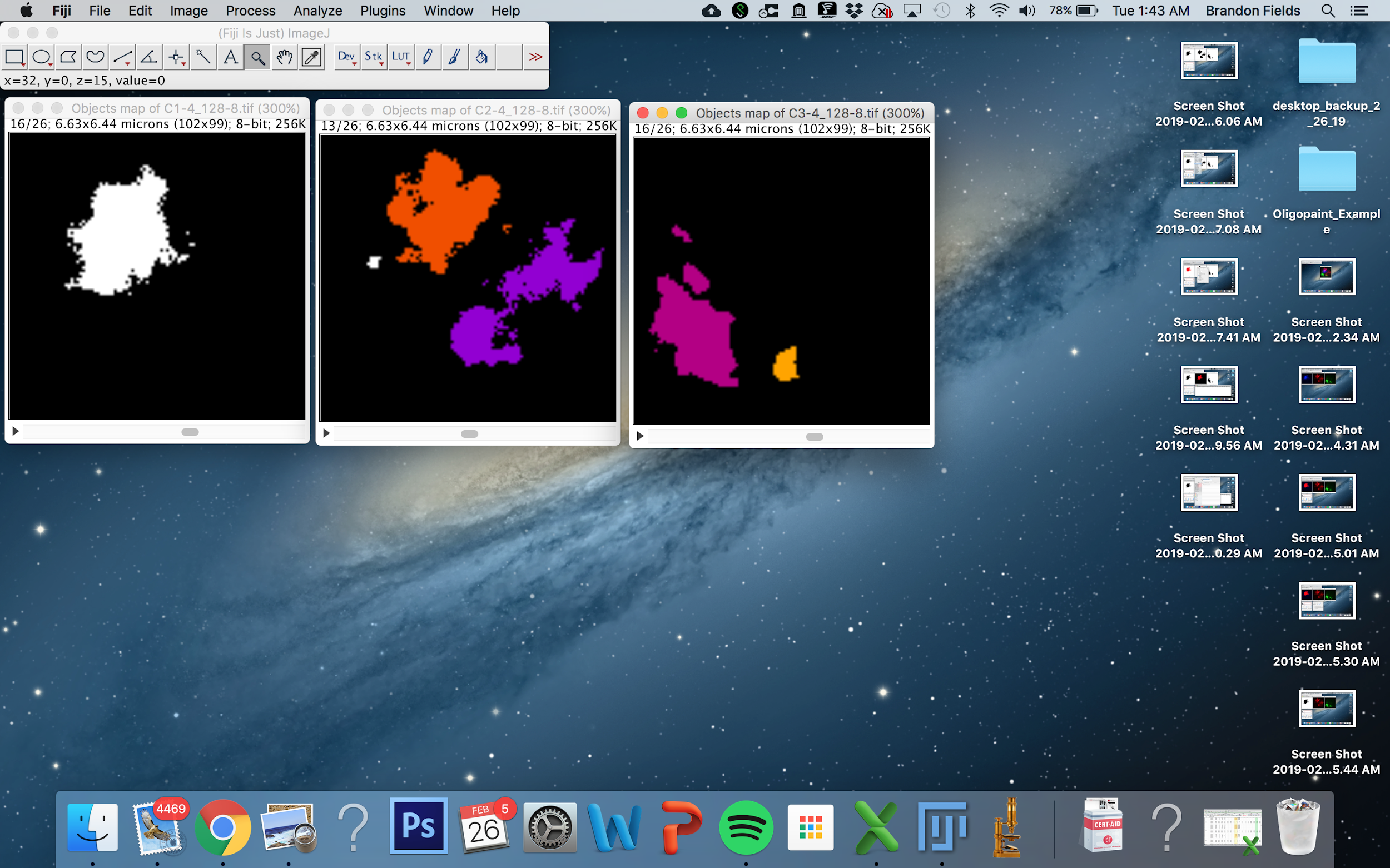


11. Select the first channel’s object map:


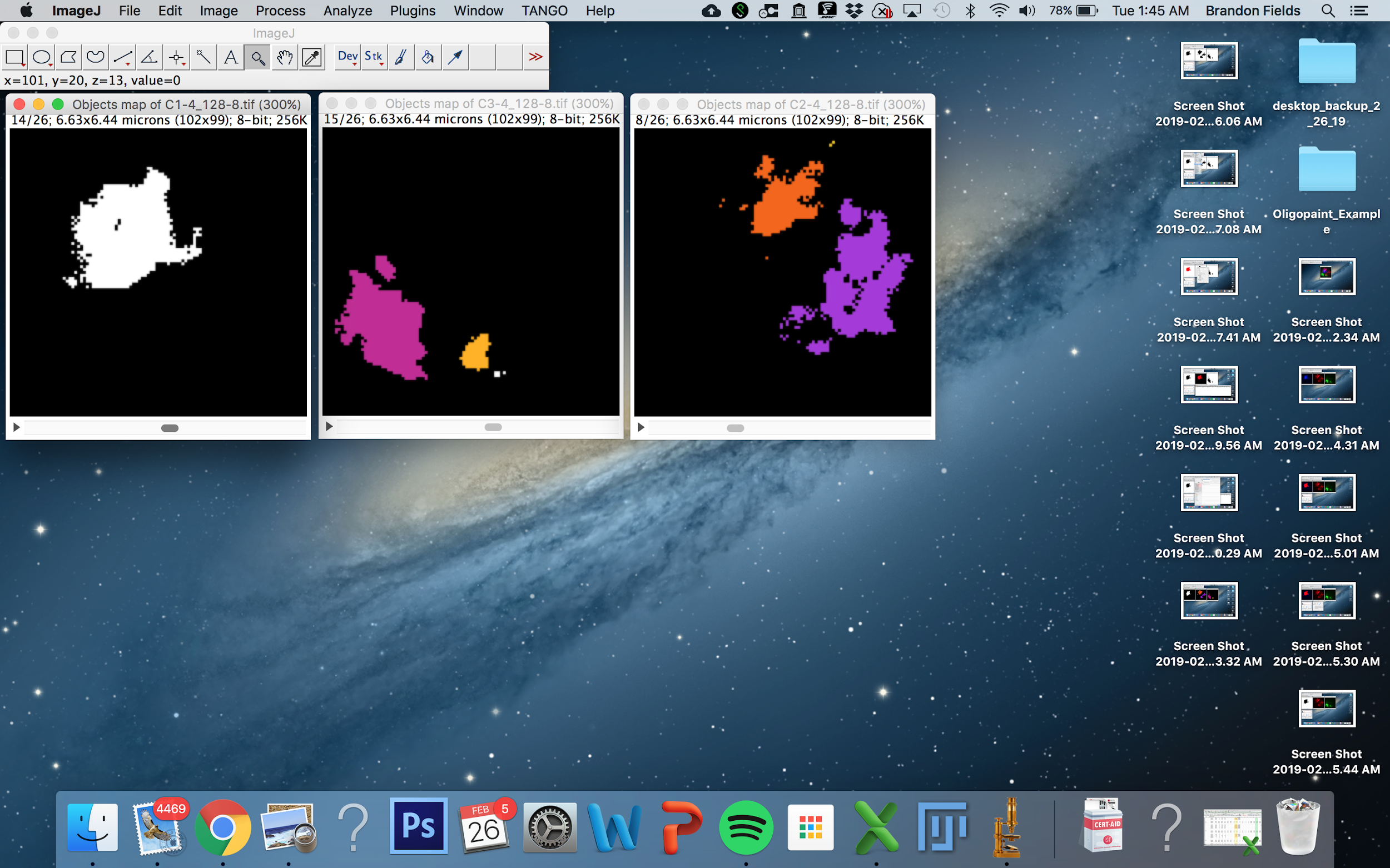


12. Open the 3D manager plug in (Plugins > 3D > 3D Manager):


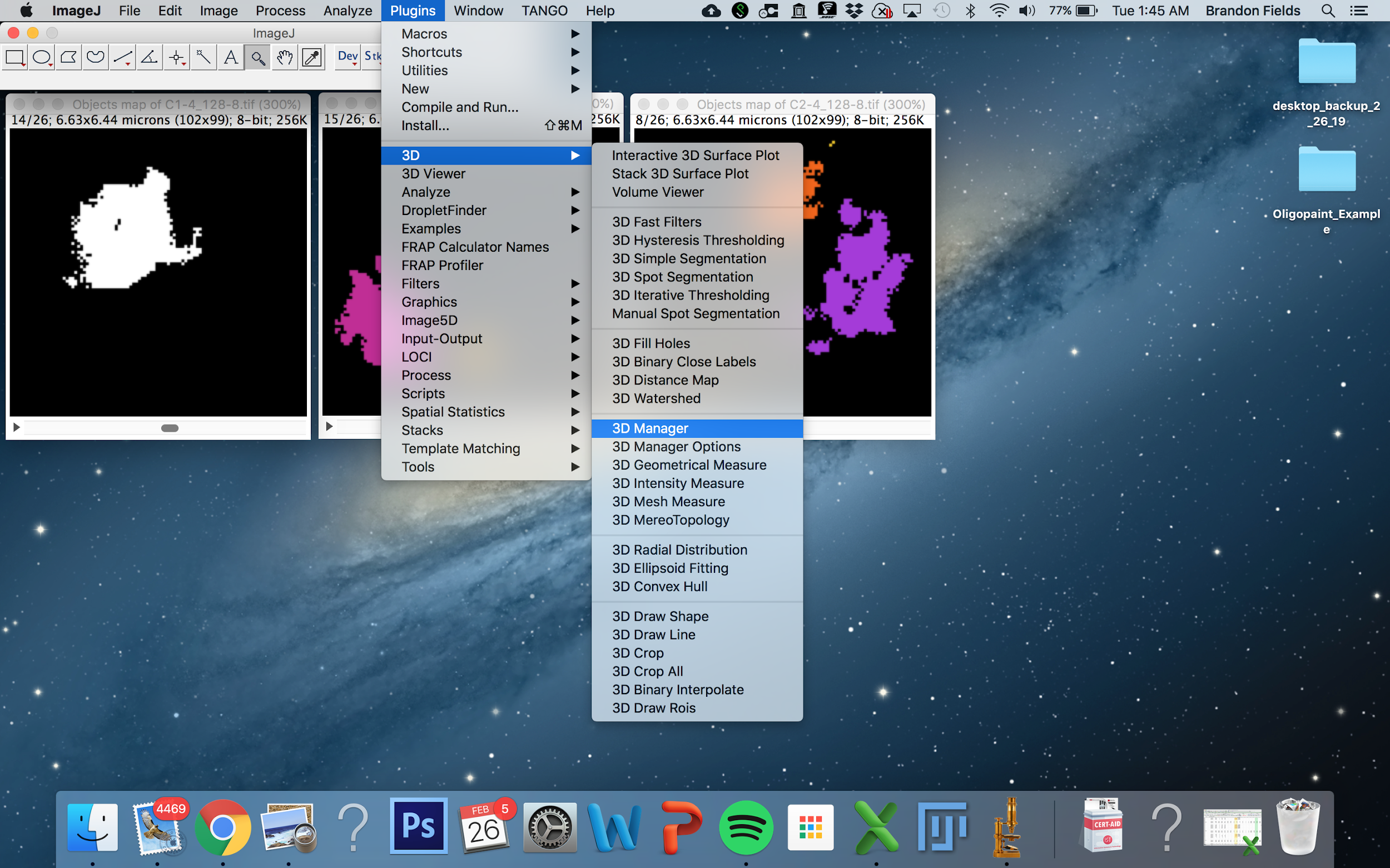


13. With the first channel image file highlighted press “Add image” to add the objects into 3D Manager (note that channel 1 has a single contiguous signal resulting in a single object (obj1)):


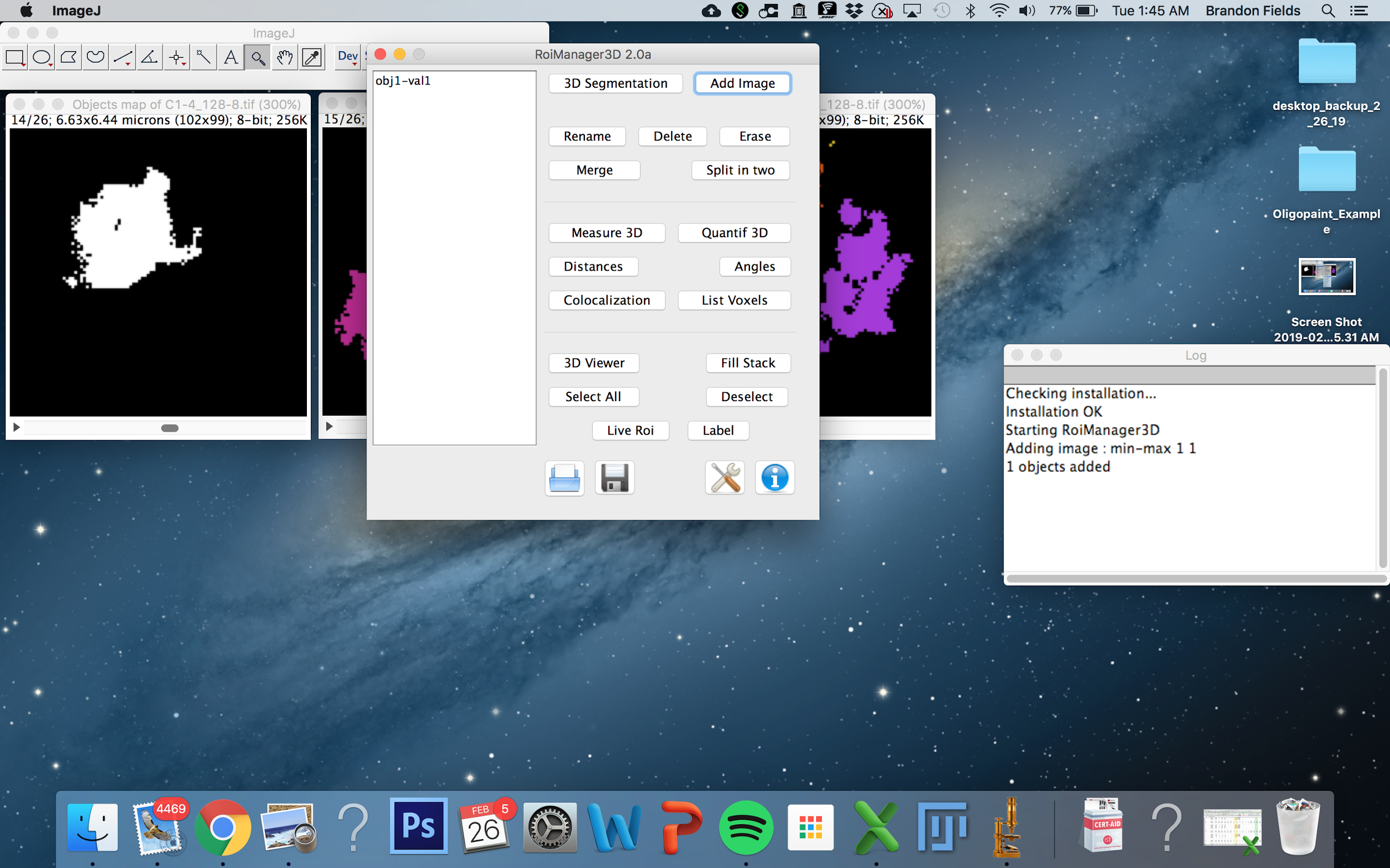


14. Next add the second channel by pressing “Add image” with the second objects file selected. Note that the 2nd channel has 4 distinct objects (val1, 2, 3, 4). These must be merged into a single object to represent the entire chromosome territory. Highlight all 4 objects and press “Merge”:


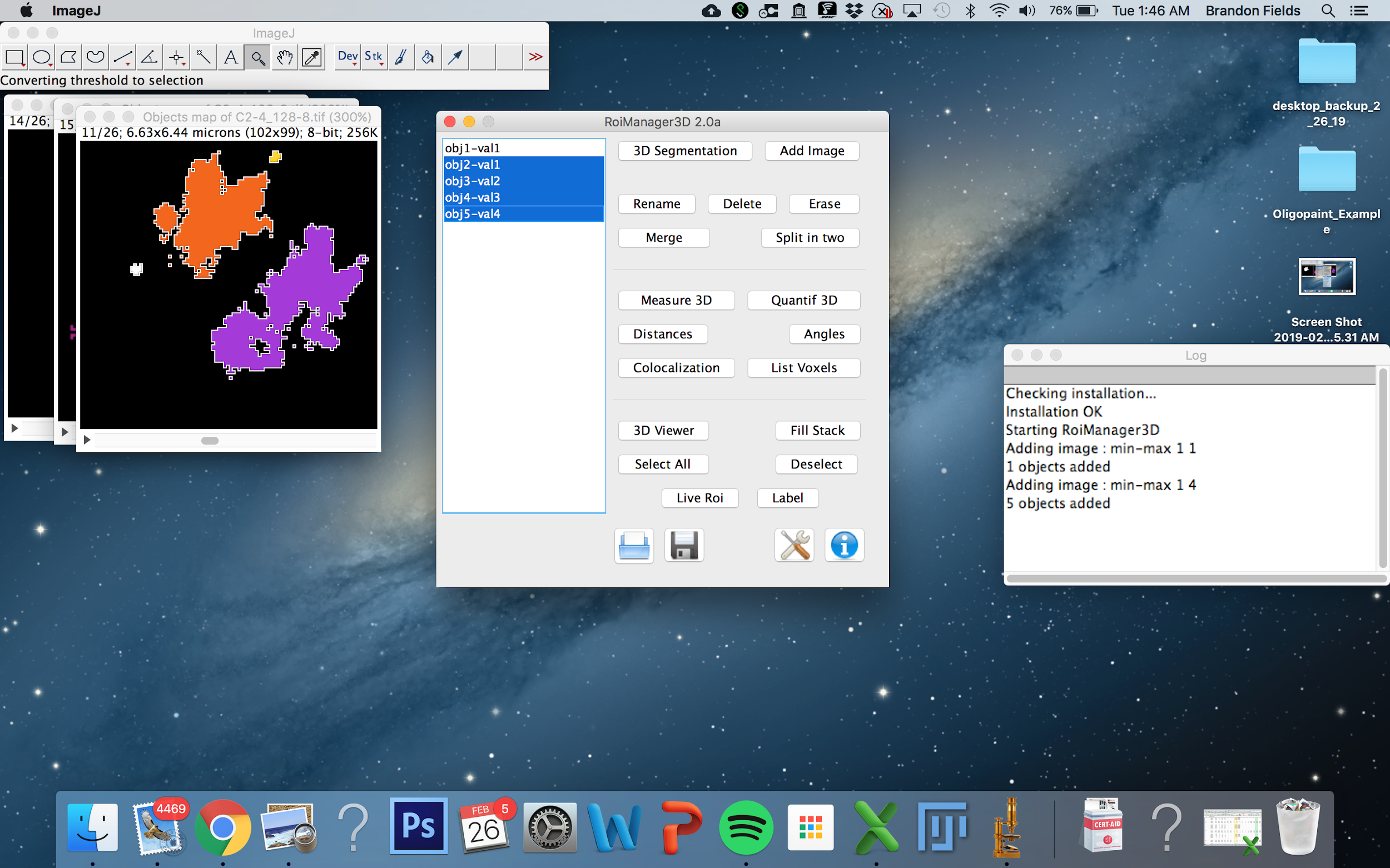


15. The result of step 14 will be a single object encompassing all 4 objects:


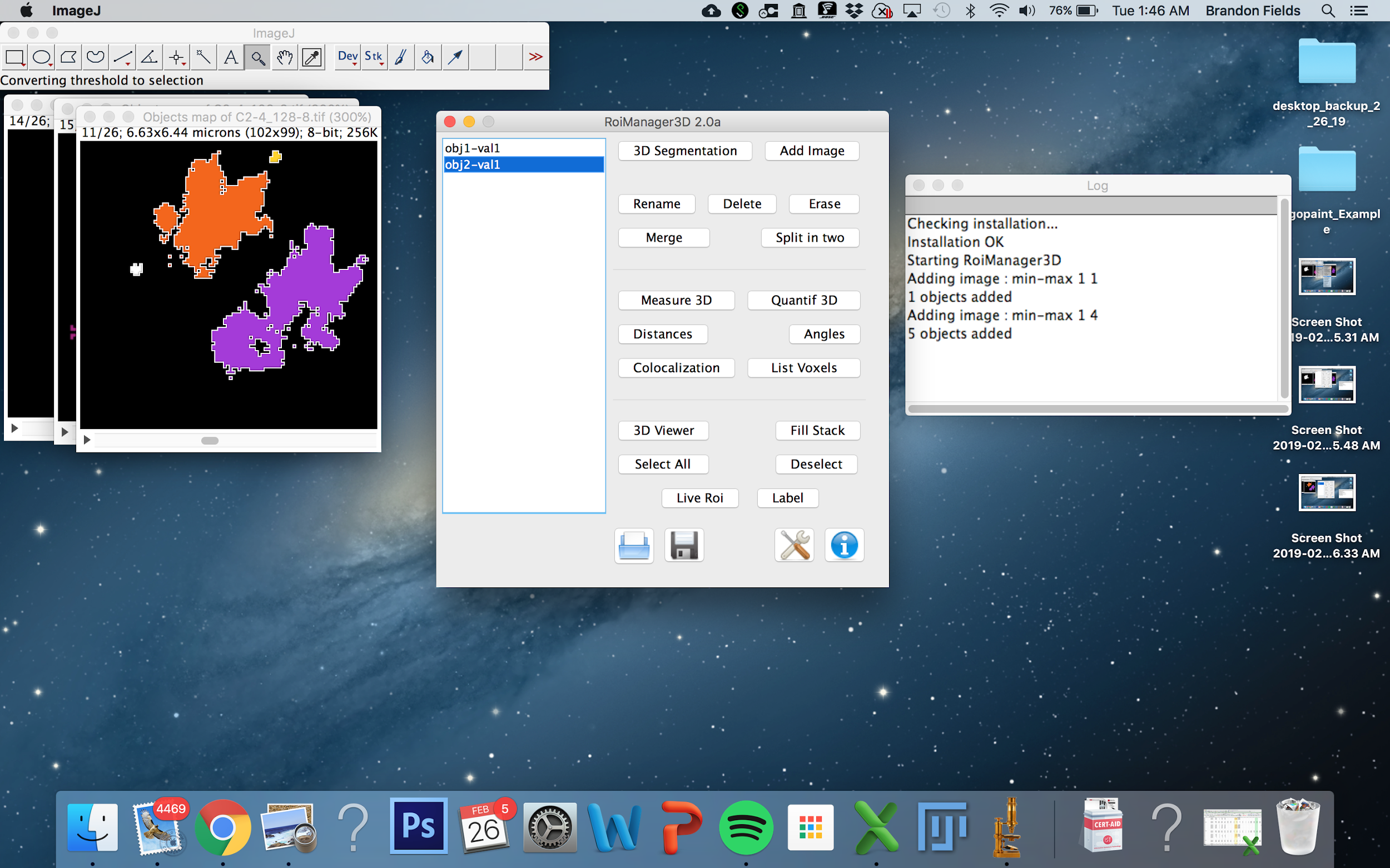


16. Repeat steps 14-15 for channel 3:


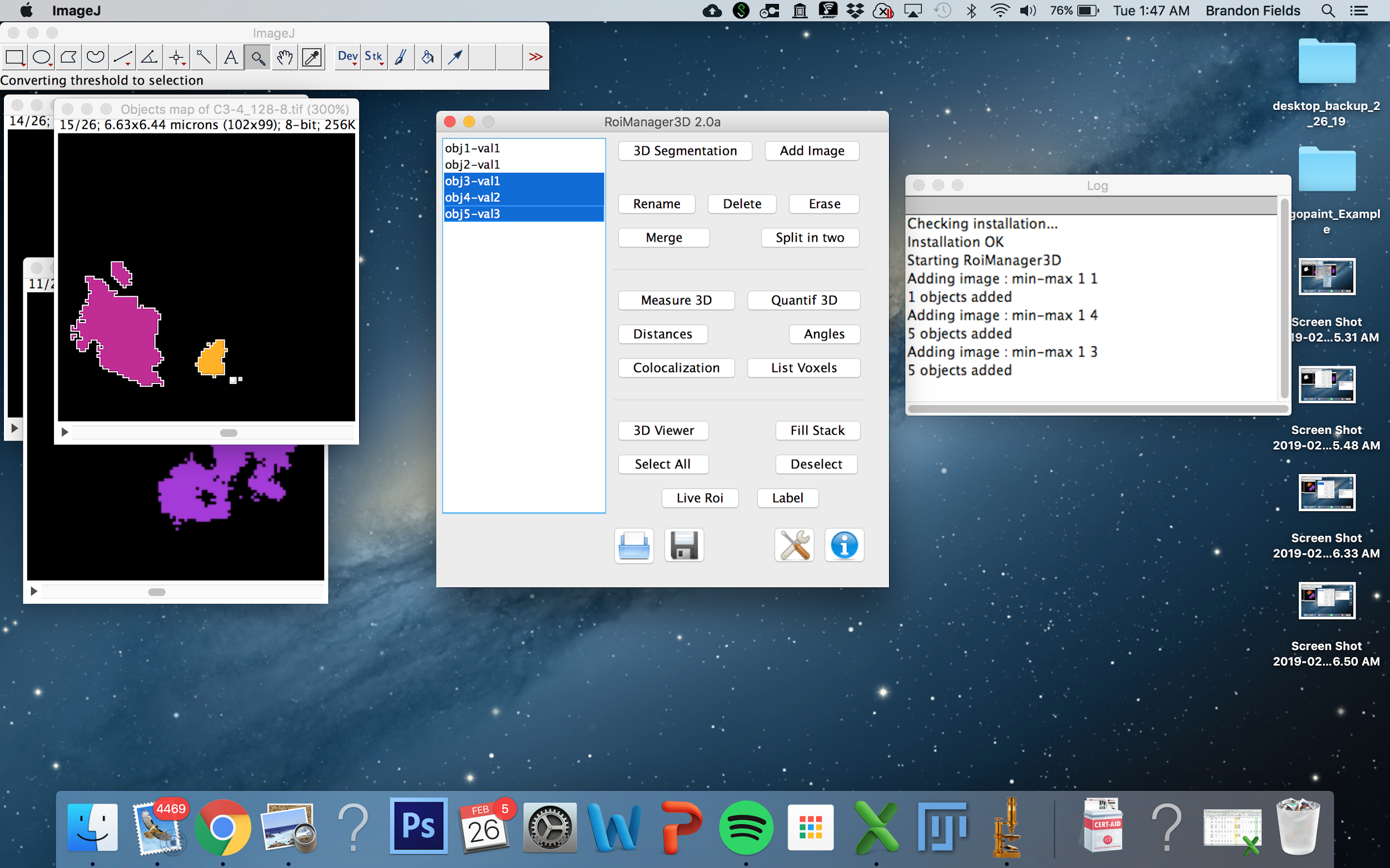


17. Highlight all three objects (note that each object is outlined on the overlay to the left):


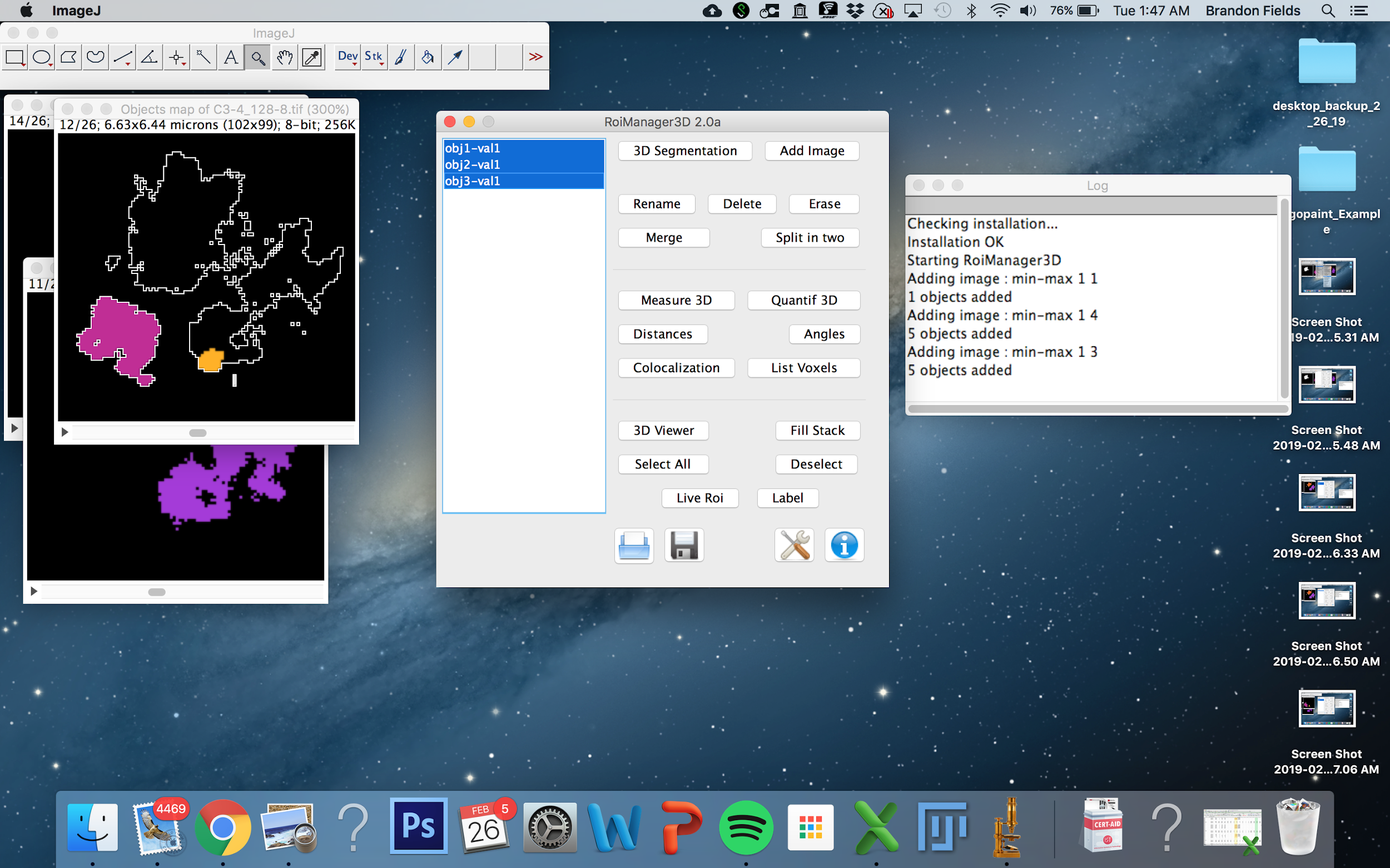


18. Calculate the percent of chromosome territory overlap between each channel by pressing the “colocalization” button with all 3 selected:


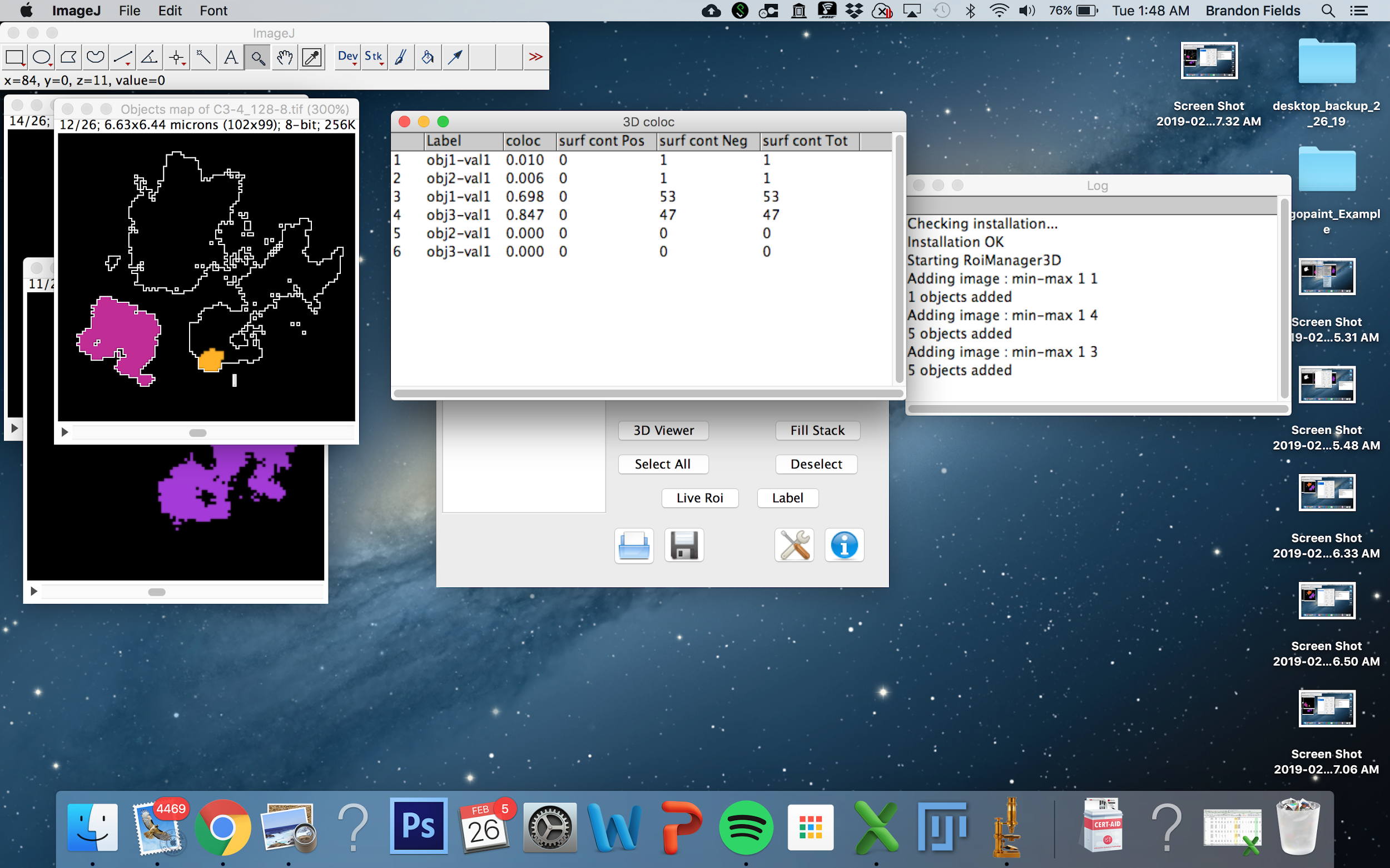


19. Copy and paste the table to excel:


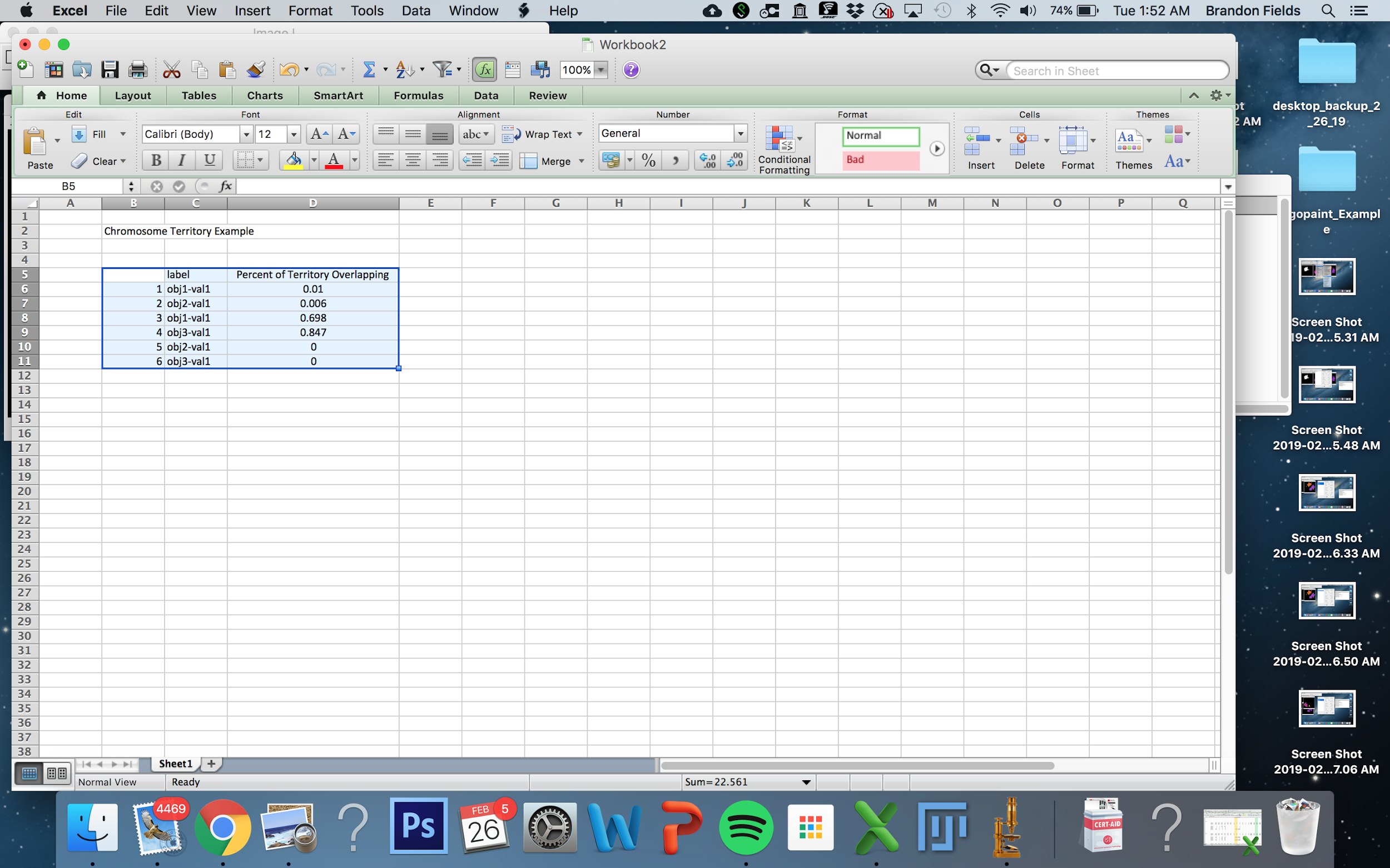


20. Next we need to calculate the volume of each territory to determine the volume of overlap (using the percentage calculated above). Or one can just calculate the volume of each territory if that is the metric you are interested in. To do so press “Measure 3D” with all object highlighted:


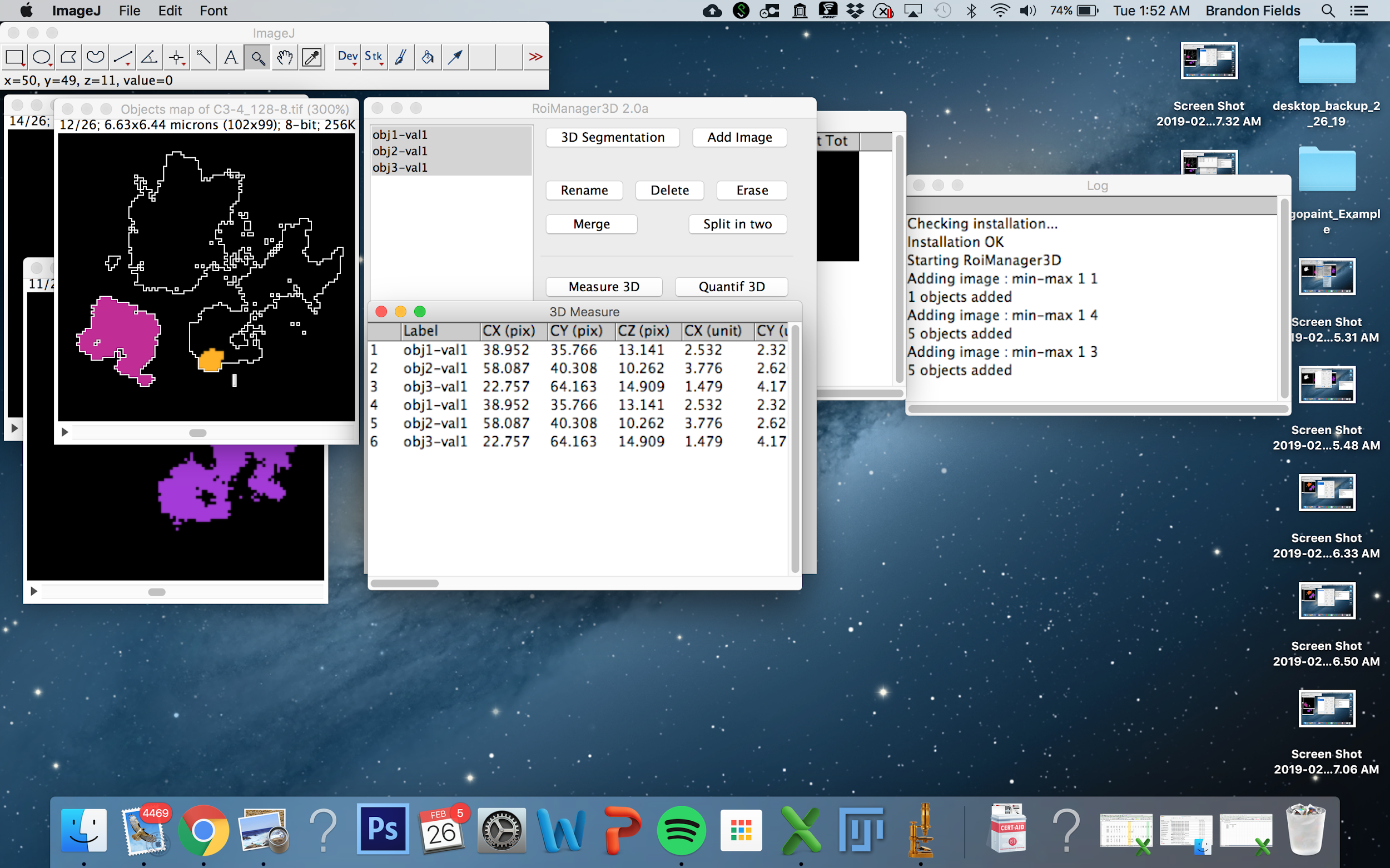


21. Copy and paste table into excel:


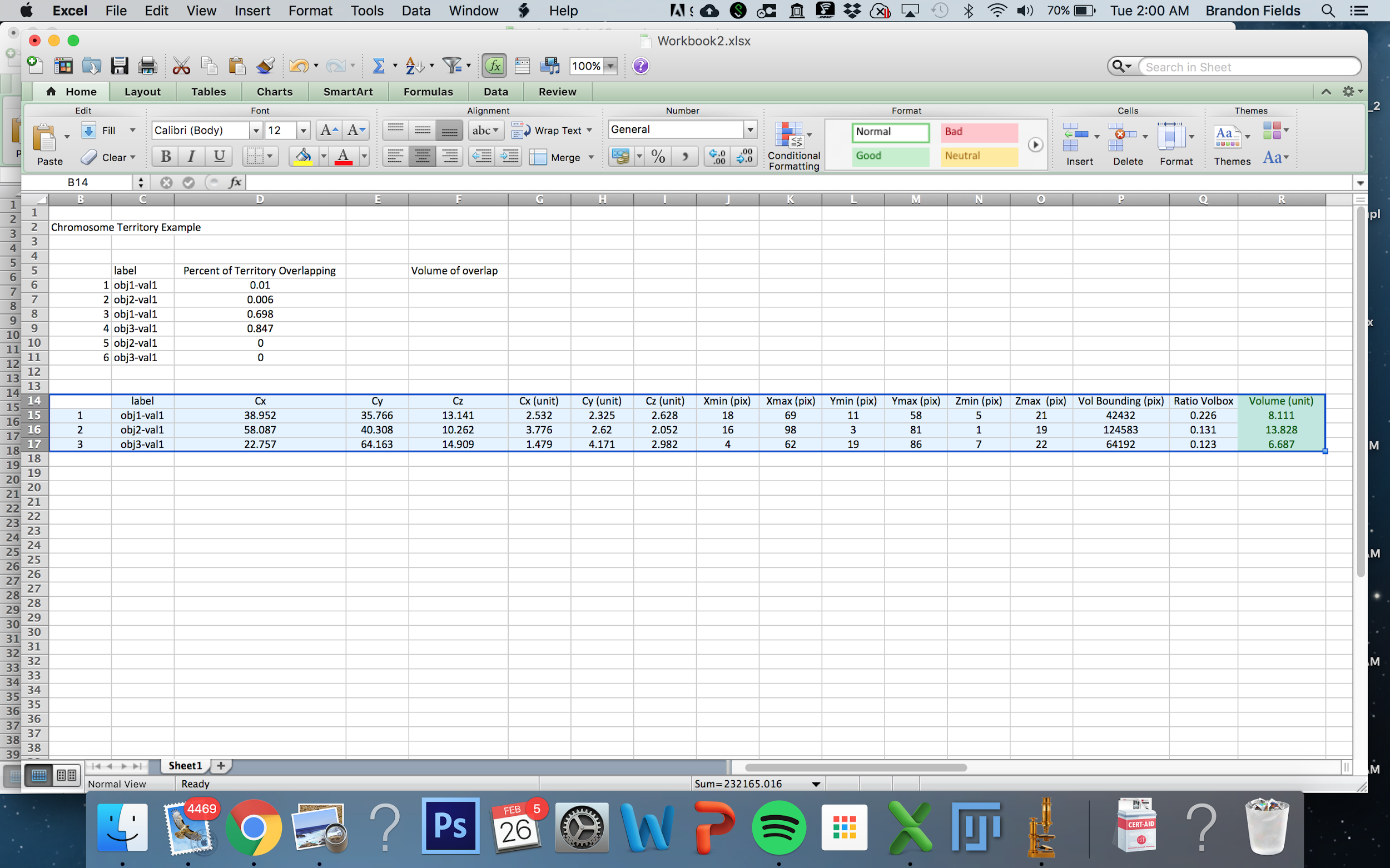


22. Multiply the percent of territory overlap by the volume of the territory:


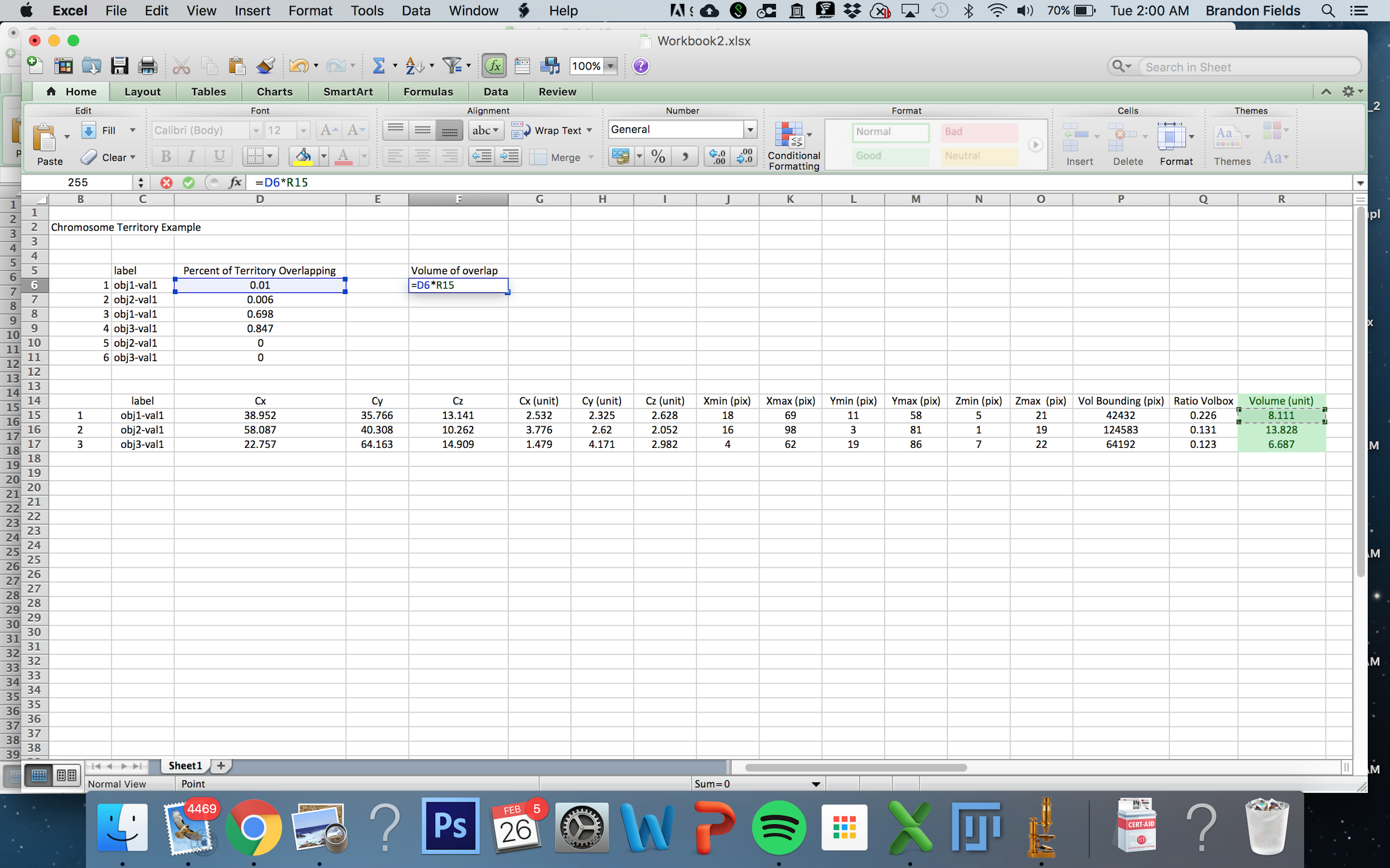


23. Repeat for each chromosome interaction:


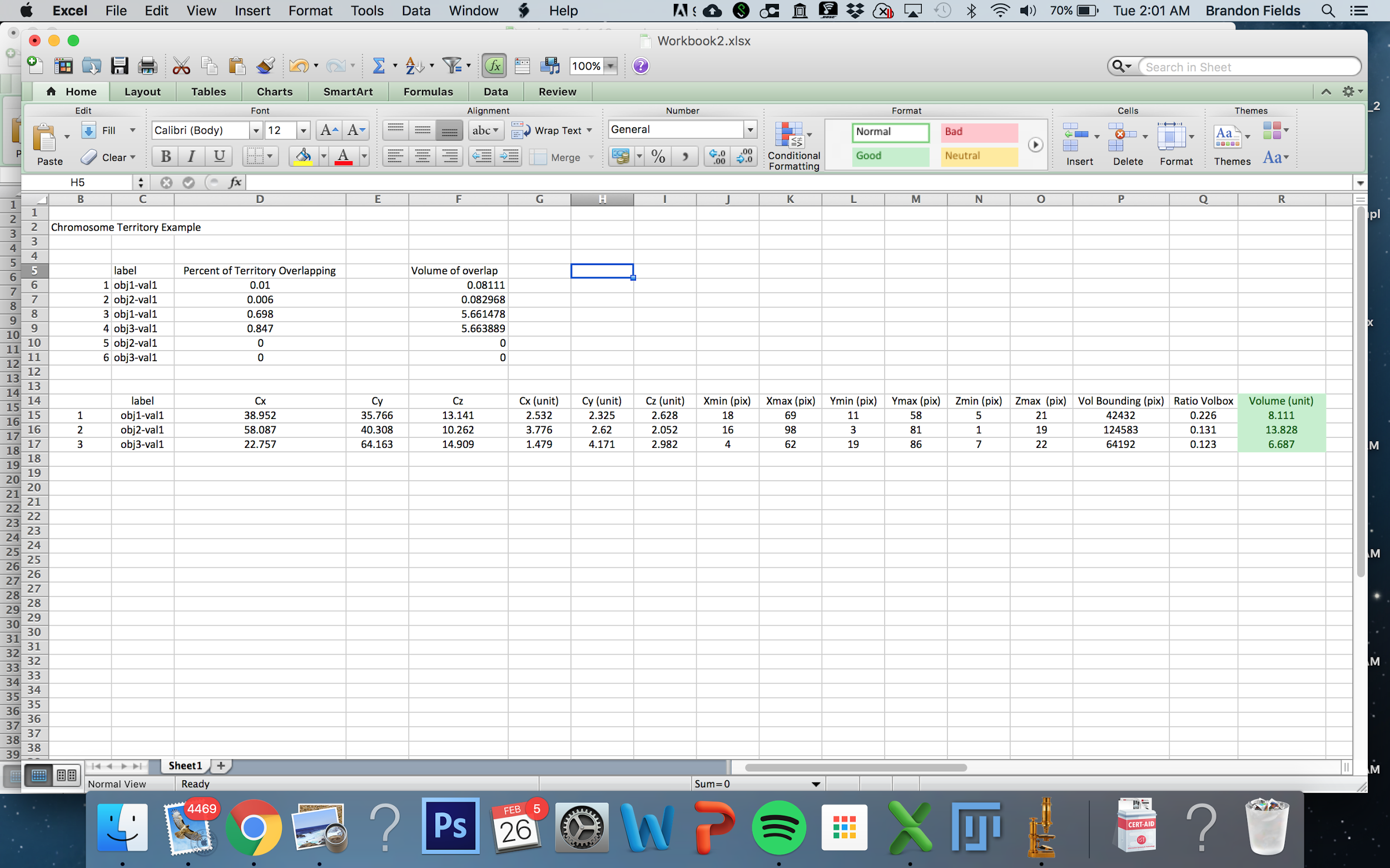


24. Divide each volume by 100 (since we multiplied by percent overlap) to determine the volume of overlap (um^3):


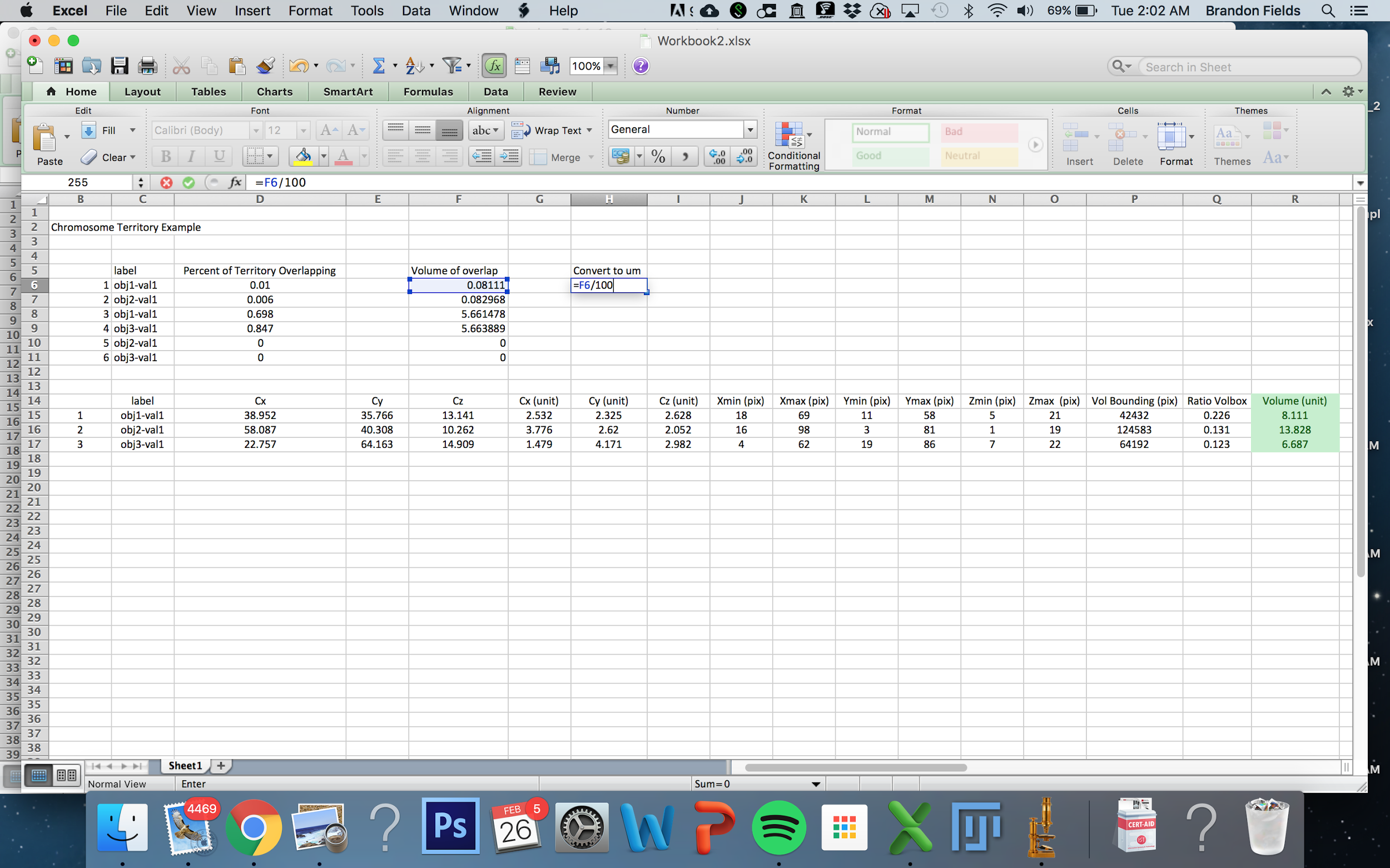


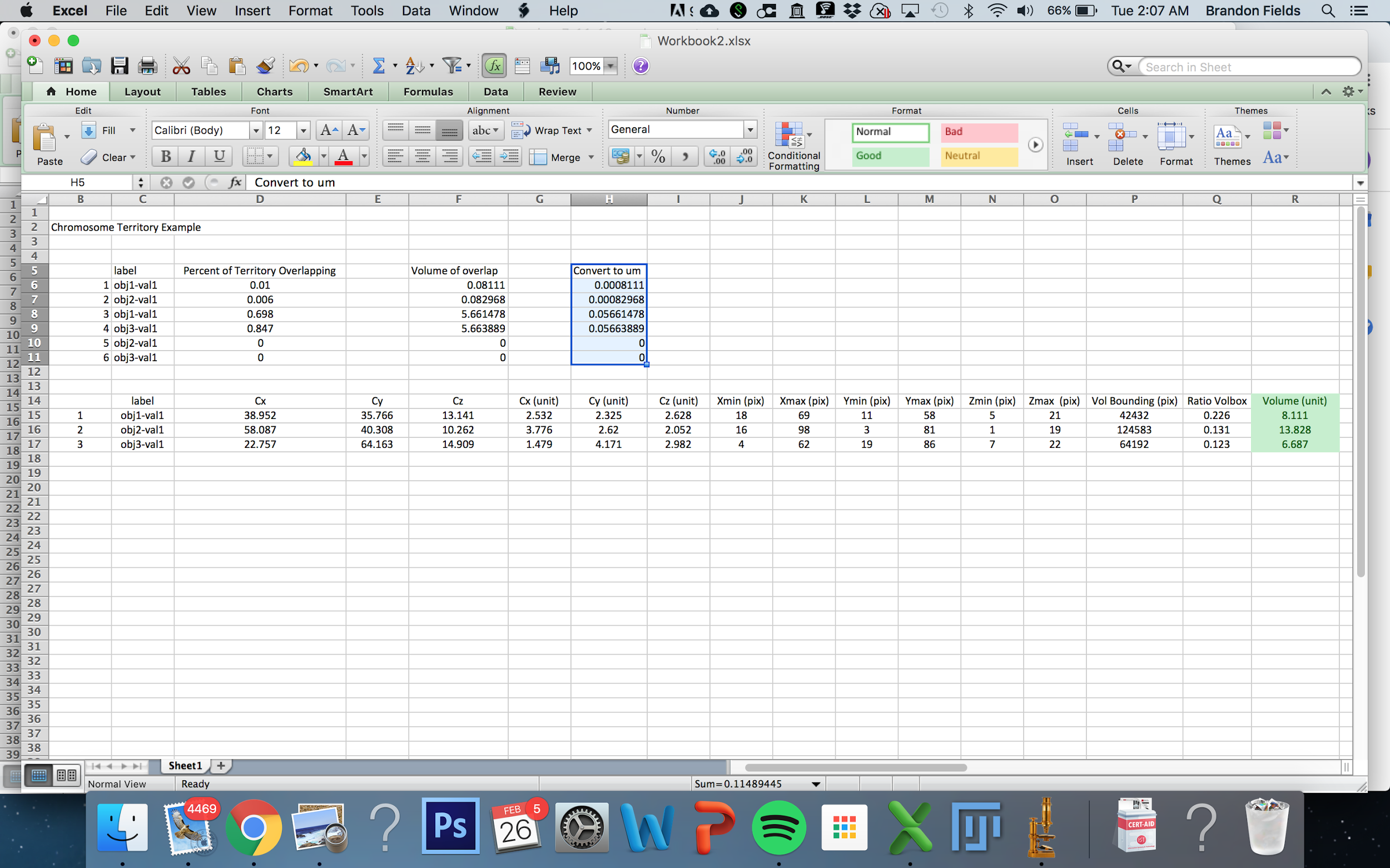


25. The result is the volume of overlap between all 3 objects:


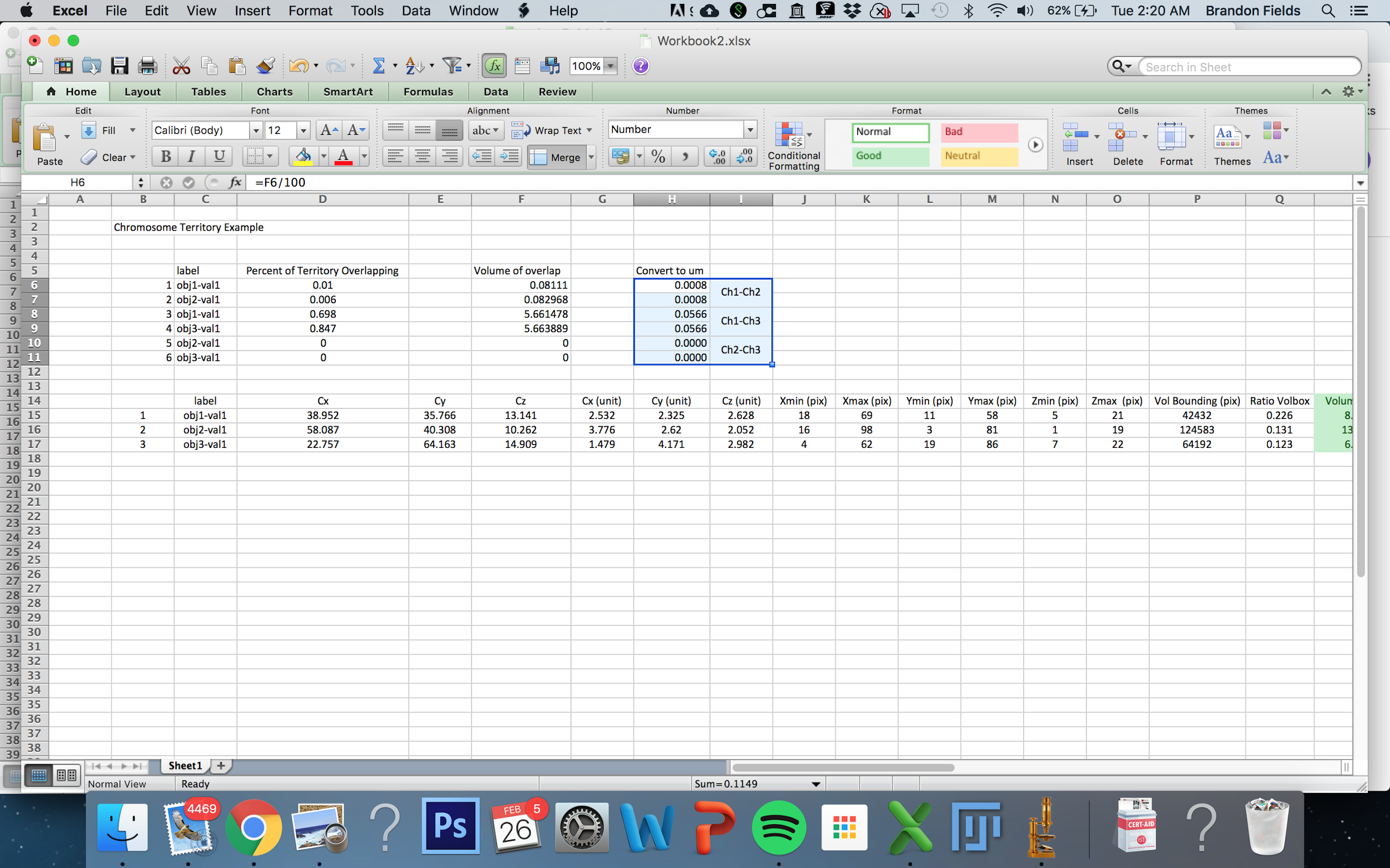

Supplement: Supplementary file 8. [file elife-42823-supp8.docx]
